# Assessment of brain response in operators subject to recoil force from firing long-range rifles

**DOI:** 10.3389/fbioe.2024.1352387

**Published:** 2024-02-14

**Authors:** Tanvi Seeburrun, Michael C. Bustamante, Devon C. Hartlen, Austin Azar, Simon Ouellet, Duane S. Cronin

**Affiliations:** ^1^ Department of Mechanical Engineering, University of Waterloo, Waterloo, ON, Canada; ^2^ Valcartier Research Centre, Defence Research and Development Canada, Quebec, QC, Canada

**Keywords:** mild traumatic brain injury, brain strain, sub-concussive injury, recoil force, finite element head model, instrumented mouthguards, 0.50 caliber rifles, repeated exposure

## Abstract

Mild traumatic brain injury (mTBI) may be caused by occupational hazards military personnel encounter, such as falls, shocks, exposure to blast overpressure events, and recoil from weapon firing. While it is important to protect against injurious head impacts, the repeated exposure of Canadian Armed Forces (CAF) service members to sub-concussive events during the course of their service may lead to a significant reduction in quality of life. Symptoms may include headaches, difficulty concentrating, and noise sensitivity, impacting how personnel complete their duties and causing chronic health issues. This study investigates how the exposure to the recoil force of long-range rifles results in head motion and brain deformation. Direct measurements of head kinematics of a controlled population of military personnel during firing events were obtained using instrumented mouthguards. The experimentally measured head kinematics were then used as inputs to a finite element (FE) head model to quantify the brain strains observed during each firing event. The efficacy of a concept recoil mitigation system (RMS), designed to mitigate loads applied to the operators was quantified, and the RMS resulted in lower loading to the operators. The outcomes of this study provide valuable insights into the magnitudes of head kinematics observed when firing long-range rifles, and a methodology to quantify effects, which in turn will help craft exposure guidelines, guide training to mitigate the risk of injury, and improve the quality of lives of current and future CAF service members and veterans.

## 1 Introduction

mTBI may occur when the head experiences a direct or indirect impact, resulting in impaired function (Schmitt et al., 2019). Symptoms of mTBI range from headaches, dizziness, and sensitivity to noise, to more severe ones such as loss of consciousness. mTBI may also lead to long-term neurological damage, including chronic traumatic encephalopathy (Harmon et al., 2013). Such symptoms have also been linked to repetitive sub-concussive incidents in contact sports athletes (Crisco et al., 2010; Stern et al., 2011; McKee et al., 2013; Meaney et al., 2014). Similarly, volunteers in the CAF have reported chronic mTBI symptoms. It has been hypothesized that a possible source of repeated exposure is from the recoil force of long-range rifles incurred during training and active duty (Cardinal et al., 2018; Adams, 2019). These types of chronic injuries have the potential to significantly reduce one’s quality of life and place a considerable burden on society (Schmitt et al., 2019). For example, in the advanced stages of CTE, affected individuals may experience symptoms such as depression, dementia, speech abnormalities, and an increased risk of suicide (McKee et al., 2013). While the long-term effects of mTBI have been studied extensively in athletes, military personnel represent an under studied population. To that end, there exists a need to understand how the repeated exposure to recoil may lead to brain injury, such that measures can be taken to avoid long-term negative outcomes (Cardinal et al., 2018).

A key aspect in assessing brain injury risk involves measuring in-field kinematics to establish a correlation between external loads (e.g., acceleration, impact force, duration) experienced during potentially injurious scenarios and the subsequent mechanical response of the brain (e.g., stress, strain) (Meaney et al., 2014). Over the past decade, a variety of wearable impact sensors have been developed, including skin-mounted (Kim et al., 1993), earplug (Knox, 2004; Sandmo et al., 2019), helmet-mounted (Beckwith et al., 2012; Allison et al., 2014), headband (Huber et al., 2021), and mouthguard (Camarillo et al., 2013; Bartsch et al., 2019) sensors, each tailored to specific sports or scenarios. The accuracy of each sensor relies on the level of coupling with skull motion, as well as the proper wearing of the sensors. The Head Impact Telemetry System (HITS), a football helmet-mounted accelerometer, remains the most commonly used sensor for collecting head impact data in the sports-environment (Wu et al., 2016; Rowson and Duma, 2011). Recent studies have indicated that custom-fit mouthguards exhibit better accuracy, possibly due to their improved coupling with the individual’s upper dentition (Wu et al., 2016; Rich et al., 2019).

Various head injury metrics for assessing the severity of potential head injuries are based on the resulting head kinematics upon impact, which can be derived from translational motion, rotational motion, or a combination of both. Initially, head injury metrics were established based solely on the linear acceleration of the head and have been extensively used to regulate safety in the sports and automotive field (Schmitt et al., 2019). For instance, the National Operating Committee on Standards for Athletic Equipment (NOCSAE) evaluates protective headgear for some sports using the Severity Index (Gadd, 1966) and the National Highway Traffic Safety Administration employs Head Injury Criterion (HIC) (Versace, 1971) for automotive safety regulations. However, there is still some uncertainty regarding the effectiveness of translational-based metrics towards predicting the relative severity of head injury, despite their extensive use (Gabler et al., 2016).

Holbourn (1943) proposed that significant brain deformation occurs primarily in shear as a result of rotational kinematics, rather than solely due to linear head motion. This behaviour is attributed to the high bulk modulus of the brain relative to its low shear modulus. Other studies (Kleiven, 2007; Kleiven, 2013; Hajiaghamemar et al., 2020; Carlsen et al., 2021) have supported this hypothesis, highlighting the importance of considering rotational kinematics when evaluating injury risk. Over time, various rotational-based metrics, such as DAMAGE (Gabler et al., 2019) and BrIC (Takhounts et al., 2013), have been introduced. Additionally, studies have shown that the tolerance to acceleration is highly dependent on the anatomical direction, with coronal rotation producing the most severe injuries (Patton et al., 2013; Hernandez et al., 2015). These findings emphasize the importance of considering both the directional components and magnitude of rotational acceleration when measuring head rotation. Nonetheless, there is currently no consensus on a universal kinematic-based injury metric that accurately predicts concussions or provides a clear correlation between kinematic measures and the occurrence of mTBI.

Alternatively, human body modelling (HBM) has become an increasingly popular and promising approach for quantifying brain deformation using intracranial parameters such as stress or strain. HBMs provide a means to better understand brain deformation and localize injury. Multiple studies (Kleiven, 2007; McAllister et al., 2012; Smith et al., 2015; Rowson and Duma, 2022) have utilized FE models to assess brain strain response during impacts, aiming to establish injury criteria and thresholds based on computational models. The Global Human Body Models Consortium (GHBMC), an extensively validated HBM including a detailed head model (Mao et al., 2013), is one such example.

Maximum principal strain (MPS), often reported as 95^th^ percentile MPS (MPS_95_), has been commonly used as deformation-based injury risk metric (McAllister et al., 2012; Patton et al., 2013) that exhibits a stronger correlation with concussion compared to kinematic metrics (Camarillo et al., 2013; Hernandez et al., 2015). However, there is no consensus on threshold values for MPS_95_ for mTBI prediction. The existing literature suggests a wide range of reported strains for concussive head injury, ranging from 0.06 to 0.448 (Rycman et al., 2023). Furthermore, multiple studies have provided evidence that specific deep inner structures of the brain, such as the corpus callosum, thalamus, brainstem, and midbrain, offer greater predictability for concussions compared to other brain regions (Viano et al., 2005; Patton et al., 2013; Hernandez et al., 2015; Patton et al., 2015). These findings suggest that mTBI thresholds may be region dependent. It is important to note that these findings were based on multiple FE models and diverse populations. In fact, Ji et al. (2014) demonstrated that the predicted strain for the same kinematic input may vary among different FE models.

One drawback of MPS_95_ is that it solely reports the occurrence of the maximum strain, which can be limited to a single location or a small volume within the brain. Consequently, this single value metric does not capture the overall strain distribution throughout the brain volume. In contrast, the cumulative strain-volume (CSV) curve has been proposed as a more comprehensive approach that considers the strain distribution across the brain volume without relying on a predetermined threshold. This method, as reported by Rycman et al. (2023), provides a more representative measure of brain tissue response and injury risk.

The aim of the study was to quantify the effects of long-range rifle recoil force exposures on CAF operators. Experimental head kinematics data was collected from CAF volunteers using instrumented mouthguards while firing three different configurations of long-range rifles, one of which had an RMS mounted on the buttstock. The measured head kinematics were then used as inputs to a FE head model to assess brain deformation using MPS_95_, a common tissue-based injury metric, and CSV, a more recent strain-based metric. Head injury criterion (HIC_15_) and brain injury criterion (BrIC), common kinematic-based metrics, were also calculated using the measured head kinematics to contextualize the injurious level of each impact within the current literature. The four metrics were used to compare the differences between the long-range rifles as well as between the volunteers.

## 2 Methods

**FIGURE 1:**
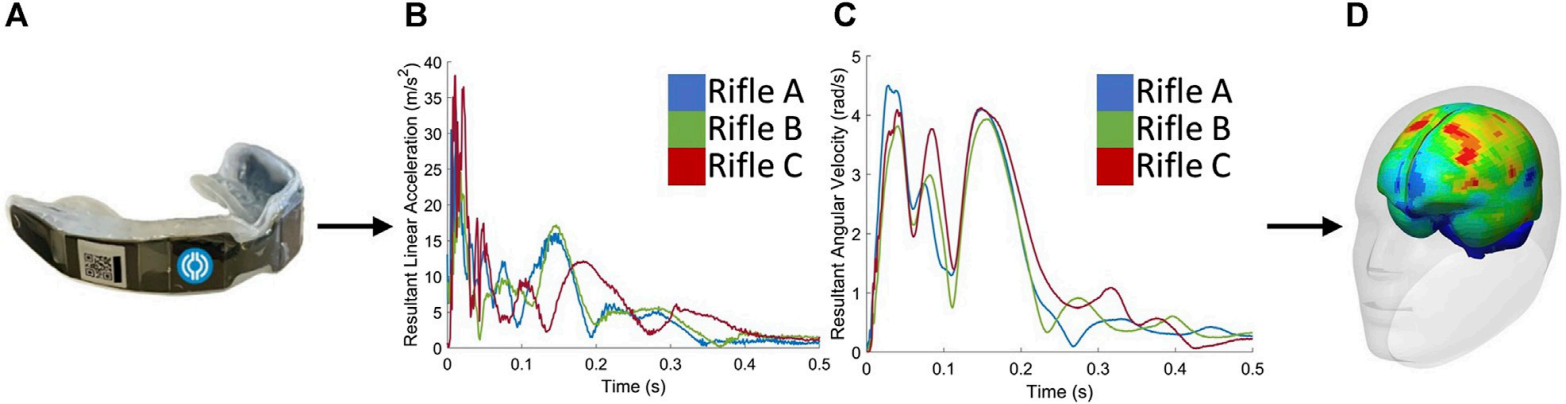
The procedure begins with **(A)** collecting data using instrumented mouthguards. Next, the measured head kinematics, including **(B)** linear accelerations and **(C)** angular velocities, are used as inputs to the **(D)** GHBMC 50^th^ percentile male head model to extract strains and quantify brain deformation.

The head kinematics of three volunteers were experimentally measured using instrumented monitoring mouthguards (IMM) (Figure 1) while they fired three different rifles. The measured head kinematics were then used as inputs to an isolated head model, where four metrics were calculated: HIC_15_, BrIC, MPS_95_, and CSV. These metrics were used to compare the strains seen by each volunteer when firing three different rifles.

### 2.1 Experimental data collection and post-processing

Experimental trials were carried out at Valcartier Research Centre, a part of DRDC, under ethics approval from the Health Research Ethics Committee (HREC-2021-009). Three experienced snipers from the CAF, with varying anthropometrics, volunteered to fire three long-range rifles, labelled A, B, and C in this study. All three rifles were 0.50 caliber rifles and used a suppressor. Rifles A and B shared the same chassis, while rifle B was equipped with an additional recoil mitigation system (RMS). Rifle C had a different chassis and no RMS. All shots were taken from the prone position, with the rifle positioned on a bi-pod at the front end of the rifle and a malleable support under the buttstock. In this posture, each volunteer was lying on their stomach behind the rifle, with their cheek rested against the buttstock, in order to use the telescopic sight. Table 1 shows the number of shots per rifle for each volunteer. The number of repeats were limited to minimize exposure of volunteers. Note that four shots were recorded for Volunteer 2 using rifle A. However, only two of these shots were considered, while the other two were excluded because of “scope bites,” which refer to instances when the volunteer’s head makes unintended contact with the telescopic sight, resulting in unwanted head accelerations.

**TABLE 1:** Number of shots and rifle configuration fired by each volunteer during experimental testing.

Rifle Configuration	Number of shots per volunteer per rifle		
	Volunteer 1	Volunteer 2	Volunteer 3
Rifle A (chassis X + suppressor)	4	2	3
Rifle B (chassis X + RMS + suppressor)	4	4	3
Rifle C (chassis Y + suppressor)	4	4	3
Total number of shots per volunteer	12	10	9

Head kinematics were captured during the firing process using IMM (Prevent Biometrics, United States). A custom version of the mouthguard (v1.5) was used, which included specific modifications such as a low-g unit accelerometer, extended recording time, and an adjustable activation threshold. Linear accelerations (X, Y, Z) and angular velocities (X, Y, Z) of the volunteer’s head were recorded with the embedded tri-axial linear accelerometer and a gyroscope, both sampling data at a frequency of 1,600 Hz. Data recording was triggered when any individual accelerometer channel exceeded a pre-set threshold of 2.5 g, capturing a 500 ms data including 7.5 ms of pre-trigger data. Additionally, raw sensor data was retrieved from the mouthguard wirelessly through an application provided by the manufacturer (Prevent Head Impact Monitoring, Prevent Biometrics, United States). No signal processing was conducted onboard the mouthguard. Ethics approval for using the human volunteer data for analysis was obtained from the University of Waterloo Office of Research Ethics (ORE #44306).

Raw linear accelerations and angular velocities from the mouthguard were transformed from the mouthguard coordinate system to the SAE J211 coordinate system for the head. It is important to note that raw acceleration signals contained the reaction force of gravity, which had to be subtracted in order to compute derived quantities such as velocity without non-physical integration drift. A two-part subtraction process was adopted, wherein the first step was to subtract the acceleration due to gravity, as measured when the volunteer’s head was stationary prior to the recoil event. However, as the volunteer’s head rotated during a recoil impulse, a single static subtraction would not eliminate effect of gravity at all orientations.

The second step involved applying a fourth-order bandpass filter, with corner frequencies of 1 and 500 Hz, to simultaneously mitigate the changing gravity vector due to head motion under recoil and eliminate any high-frequency sensor noise. Corner frequencies were chosen based on literature values (Gellner et al., 2023) and using frequency domain analysis to eliminate noise and the changing gravity vector without impacting measured accelerations associated with recoil. A simpler fourth-order low-pass filter with a cut-off frequency of 500 Hz was applied to the angular velocity signals to eliminate noise as gravity did not influence angular velocity measurements.

After filtering, a rigid body kinematic transform was applied to determine linear acceleration and angular velocity at the head center of gravity (CoG) for implementation in the numerical head model. Linear acceleration at the head, $a_{head}$ was calculated using Eq. 1, where $a_{mg}$ were measured mouthguard linear accelerations. Angular acceleration, α , was calculated by taking the derivative of experimentally measured angular velocity, ω , using the Savitzky-Golay filter (Savitzky and Golay, 1964) with a linear polynomial and window of 7. Angular velocity, ω , the other input to the head model, did not require a kinematic transform. The distance from the mouthguard sensor location to the head CoG was, r , was selected based on anthropometrics from an average male.

$$a_{\text{head}}=a_{\text{mg}}+\alpha \times r+\omega \times \left(\omega \times r\right)$$ (1)

### 2.2 Finite element head model

**FIGURE 2:**
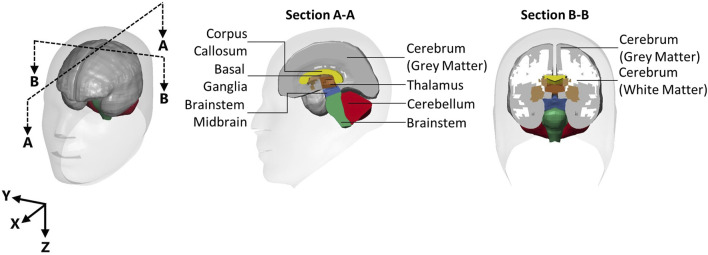
The eight brain regions in the GHBMC 50^th^ percentile male head model.

Processed head kinematics for each recoil event were used as inputs to the isolated GHBMC 50^th^ percentile male head model (version 5) based on previous work (Bruneau and Cronin, 2021). It should be noted that the GHBMC model represents an approximately 50^th^ percentile male, and the geometry of the head model was not modified for the present study. Thus, the boundary conditions were subject-specific, and they were applied to the 50^th^ percentile male finite element head model. This limitation stems from our adherence to the ethics protocol, which restricted sharing of the subject anthropometric data to protect the identity of subjects in this study, conducted with a small population. The head model was extracted from the GHBMC 50^th^ percentile male whole-body model at the foramen magnum, which included a small length (34 mm) of the spinal cord. The free end of the spinal cord was free to move, since an initial study comparing a fixed and free end showed no difference in response in the brain regions assessed in this study. The skull of the head model was treated as rigid to apply the measured linear accelerations and angular velocities to the CoG of the head model through prescribed rigid body motions, similar to previous studies (Zhang et al., 2004; Sanchez et al., 2019; Bruneau and Cronin, 2021). The kinematics from each of the 31 individual shots were modelled separately. All models were run on a high-performance computing cluster and solved using LS-DYNA (R9.2 single-, ANSYS, Canonsburg). Strain measurements were extracted for the whole brain and the eight individual regions that make up the whole brain model (Figure 2), which include the cerebellum, cerebrum gray matter, cerebrum white matter, corpus callosum, thalamus, midbrain brainstem, brainstem, and basal ganglia.

### 2.3 Metrics for brain response comparison

Four metrics were used in this study to assess severity of loading: HIC_15_ (a linear kinematic-based metric), BrIC (a rotational kinematic-based metric), MPS_95_ (a strain-based metric), and the CSV (a more recent strain-based metric).

HIC_15_ was determined by calculating the maximum area under the resultant linear acceleration-time plot over a specified time period of 15 ms, using Eq. 2.

$${\text{HIC}}_{15}=\max\left\{\left(t_{2}-t_{1}\right)\;{\left[\frac{1}{t_{2}-t_{1}}\int_{t_{1}}^{t_{2}}a\left(t\right)\text{dt}\right]}^{2.5}\right\}$$ (2)

where $a\left(t\right)$ is the resultant linear acceleration (in g) measured during the experiments, which was filtered and transformed to the head CoG as described in Section 2.1, and $t_{1}$ and $t_{2}$ (in seconds) are timepoints during the acceleration pulse selected such that the value of HIC_15_ is maximized (Schmitt et al., 2019).

BrIC was calculated using Eq. 3 where $\omega_{x}$ , $\omega_{y}$ and $\omega_{z}$ and $\omega_{xC}$ , $\omega_{yC}$ and $\omega_{zC}$ are maximum angular velocities (in rad/s), obtained from the signals measured during the experiments, which were filtered and transformed to the head CoG as described in Section 2.1, and critical angular velocities (in rad/s) about the *X*, *Y*, *Z* axes, respectively. The following critical values ( $\omega_{xC}$ = 66.30 rad/s, $\omega_{xC}$ = 53.80 rad/s and $\omega_{xC}$ = 41.50 rad/s) were used in this study (Takhounts et al., 2013).

$$\text{BrIC}=\sqrt{{\left(\frac{\omega_{x}}{\omega_{\text{xC}}}\right)}^{2}+{\left(\frac{\omega_{y}}{\omega_{\text{yC}}}\right)}^{2}+{\left(\frac{\omega_{z}}{\omega_{\text{zC}}}\right)}^{2}}$$ (3)

The MPS_95_ was used to exclude elements that have artificially high strains due to numerical artifacts (Gabler et al., 2016; Elkin et al., 2019; Bruneau and Cronin, 2021). MPS was calculated from each solid element of the 8 regions of the brain throughout time. Then the 95^th^ percentile of all the elements’ MPS were taken as the MPS_95_. Although, MPS_95_ has been widely used to assess brain response, this single value metric was limited as it only represented the maximum strain potentially occurring at one location of the brain.

**FIGURE 3:**
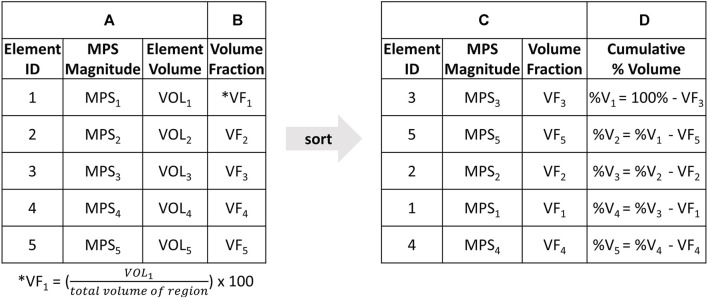
The step-by-step process to generate cumulative strain-volume curve; starting with **(A)** extracting MPS and initial volume of each element in a specific brain region during the entire simulation time, followed by **(B)** calculating the volume fraction of each element in the region as the initial volume relative to the total volume of the region. **(C)** The elements are then ordered in ascending order of MPS. **(D)** The percentage volume is calculated by subtracting the volume fraction of each element from the volume of the region as calculated in the preceding row, starting with the initial volume of 100%. **(E)** The CSV curves are plotted with **(D)** Cumulative %Volume on the Y axis and **(A)** MPS magnitude on the X axis.

The CSV approach addressed this limitation by providing a detailed representation of the strain distribution relative to the volume, without relying on percentile. The CSV curves plot the cumulative percentage volume of a region (y-axis) that experience at least a given MPS (x-axis). To generate CSV plots, the MPS and initial volume of each element in a specific region of the brain was extracted during the entire simulation time (Figure 3A). The volume fraction of each element in that region was also calculated as the initial volume of each element relative to the total volume of the region (Figure 3B). The elements were then ordered in ascending order of MPS (Figure 3C). To calculate the percentage volume, the volume fraction of each element was subtracted from the volume of the region as calculated in the preceding row, beginning with the initial volume of 100% (Figure 3D). Finally, the CSV curves were generated.

CSV plots are interpreted by correlating the amount of the volume of a region that experiences more than a given strain. For example, 100% of a volume would experience a strain greater than 0 MPS. However, the plot decreases as less and less of the volume of the brain experiences high strains. This allows the CSV plot to gauge the extent to which the entire brain experiences significant deformation. To compute a scalar summary value for statistical assessment, CSV plots were integrated with respect to percentage volume. In other words, the area under the CSV curves bounded by the y-axis (A_CSV_) was calculated. A one-way ANOVA with a significance level of *α* = 0.05 was conducted for all four metrics to assess differences in brain response between each rifle configuration and volunteer.

## 3 Results

### 3.1 Head kinematics

**FIGURE 4:**
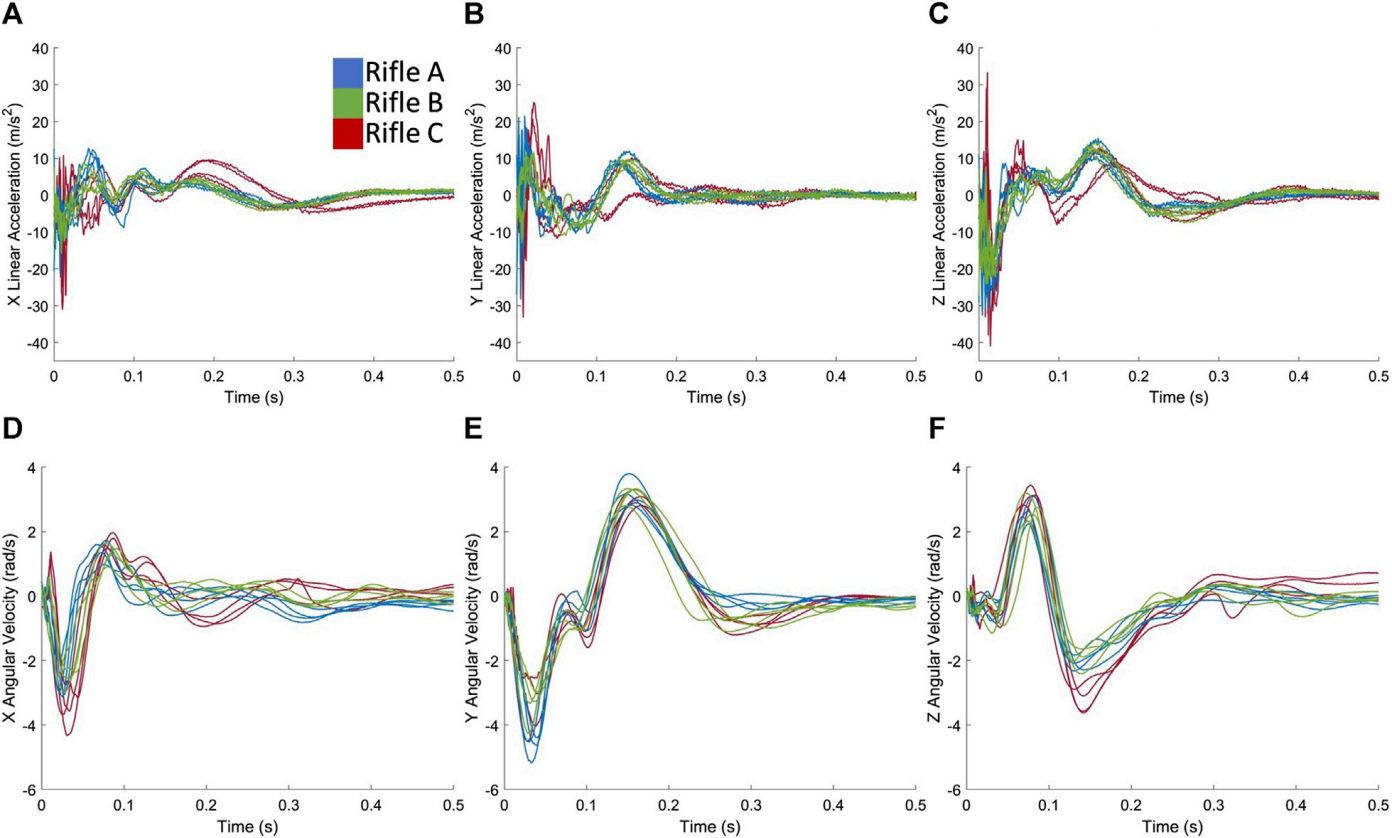
Exemplar case showing head kinematics **(A)** linear acceleration in X, **(B)** linear acceleration in Y, **(C)** linear acceleration in Z, **(D)** angular velocity in X, **(E)** angular velocity in Y, **(F)** angular velocity in Z for all shots taken by Volunteer 1 using rifles A, B and C.

The head kinematics (linear acceleration and angular velocities about the X, Y, and Z directions) of three volunteers operating the three long-range rifle configurations exhibited good inter-subject repeatability (Figure 4; Supplementary Figures S1, S2). As the rifle’s buttstock impacts the volunteer’s shoulder, the recoil forces are transmitted through the shoulder and neck, causing accelerations in the head. When the kinematic traces were grouped by rifle, the head linear accelerations and angular velocities exhibited broadly similar features. The peak accelerations occurred within the first 0.05 s. While the timing of the first peak was similar for all three rifles, the magnitudes of both the linear accelerations and angular velocities varied with rifle C typically demonstrating higher magnitudes, followed by rifle A and B.

**FIGURE 5:**
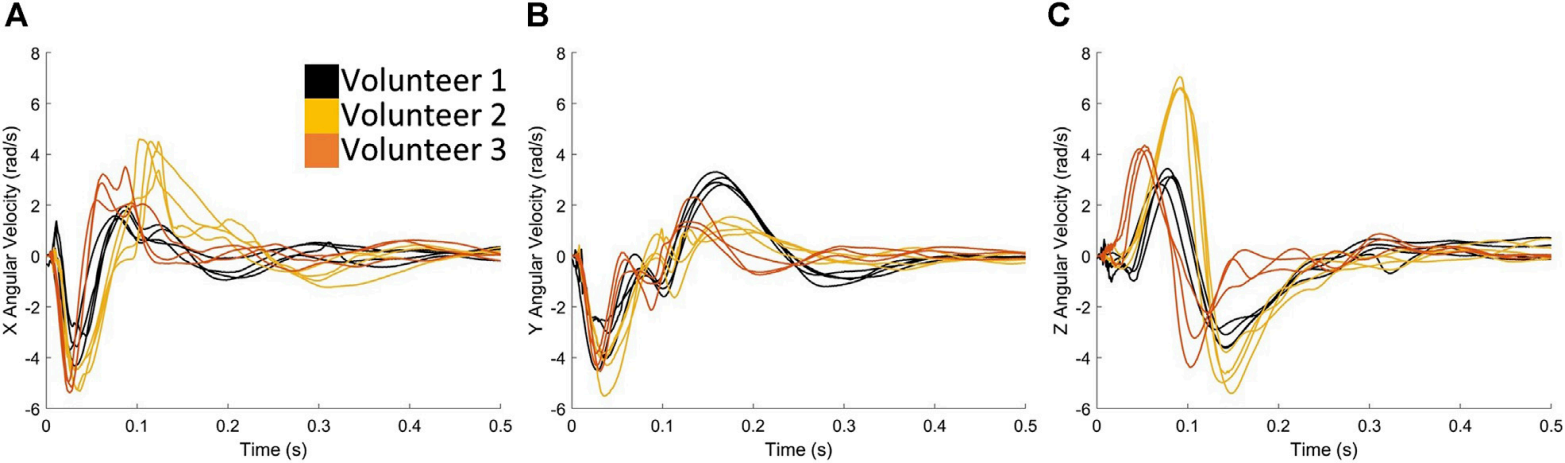
Exemplar case comparing the angular velocities **(A)** in X, **(B)** in Y and **(C)** in Z directions when volunteers are firing rifle C.

Although similar signal features were observed between rifles for each volunteer (Figures 4A–F), variation was observed between operators, particularly with respect to angular velocity (Figures 5A–C). There was relatively symmetrical motion in the angular velocity about the z-axis (axial plane) for all three volunteers, starting with a positive velocity peak followed by a negative velocity trough of lesser magnitude (Figure 5C). The peak corresponded to the rotation of the head towards the shoulder impacted by the buttstock of the rifle. The subsequent trough indicates the volunteer’s head returning back to its initial position.

Rotation in both positive and negative directions about the y-axis (sagittal plane) (Figure 5B) can be observed, suggesting forward and backwards motion of the head during the firing process. The first trough of angular velocity-time plot in the y-axis (Figure 5B) was consistent across all three volunteers, but the second peak was higher for Volunteer 1, indicating more motion in the sagittal plane compared to Volunteers 2 and 3. Head rotation about the x-axis (coronal plane) was also observed (Figure 5A), which is a known cause of severe brain injuries (Patton et al., 2013; Hernandez et al., 2015). Notably, this rotation primarily occurred in one direction (negative) for Volunteer 1, while Volunteers 2 and 3 exhibit an additional second peak, indicating more motion in the coronal plane. Overall, these observations highlight the individual variations in head motion during the firing process. Note that similar observations were found for rifles A (Supplementary Figure S3) and B (Supplementary Figure S4).

### 3.2 HIC_15_ calculation by rifle

**FIGURE 6:**
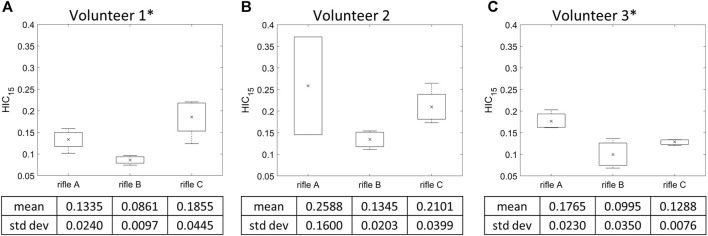
Mean HIC_15_ and variation of the three rifles for **(A)** Volunteer 1, **(B)** Volunteer 2, **(C)** Volunteer 3. * = statistically significant different detected between rifles. Number of trials for Volunteer 1 (rifles A:4, B:4, C:4), Volunteer 2 (rifles A:2, B:4, C:4) and Volunteer 3 (rifles A:3, B:3, C:3).

HIC_15_ was calculated for each shot taken by every volunteer (summarized in Supplementary Table S1), and the mean HIC_15_ values were determined for each rifle (Figure 6). It can be noted that Volunteer 1 had higher HIC_15_ values while firing rifle C, followed by rifle A and then B. However, Volunteers 2 and 3 had higher HIC_15_ values while firing rifle A compared to rifles B and C. Rifle B exhibited the lowest HIC_15_ values for all three volunteers. The standard deviation is notably higher for Volunteer 2 when using rifle, A, which is based on only two shots. The increase HIC_15_ was due to the presence of a high magnitude peak at t = 100 ms in the linear acceleration data (Supplementary Figures S1A–C). The calculation of HIC_15_ relies on determining the maximum area under the linear acceleration-time curve. In this case, the high standard deviation is a consequence of the short-duration high magnitude peak in the linear acceleration signals for one of the two shots, leading to an uncharacteristic HIC_15_ value. A one-way ANOVA analysis was conducted with a significance level of *α* = 0.05 to further analyze the differences between the rifles for each volunteer. A significant difference was observed between rifles in HIC_15_ values for Volunteers 1 and 3, with *p*-values of 0.00363 and 0.0233, respectively. However, no significant difference was detected between the rifles for Volunteer 2, with a *p*-value of 0.144.

### 3.3 BrIC calculation by rifle

**FIGURE 7:**
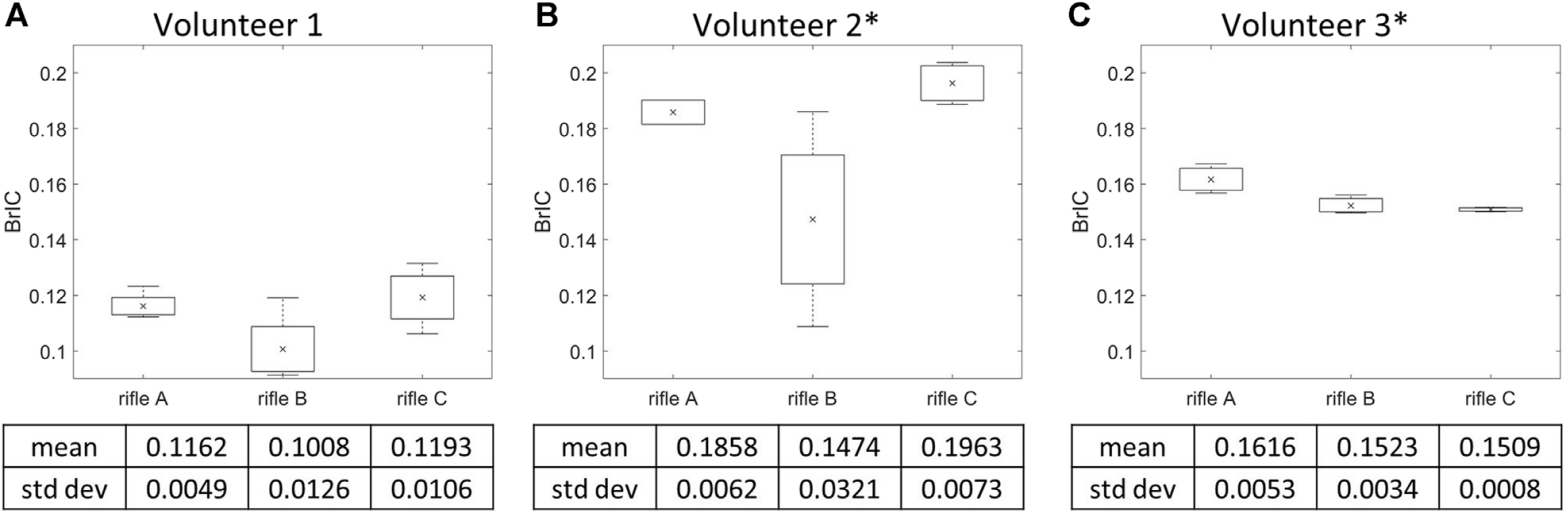
Mean BrIC and variation of the three rifles for **(A)** Volunteer 1, **(B)** Volunteer 2, **(C)** Volunteer 3. * = statistically significant different detected between rifles. Number of trials for Volunteer 1 (rifles A:4, B:4, C:4), Volunteer 2 (rifles A:2, B:4, C:4) and Volunteer 3 (rifles A:3, B:3, C:3).

BrIC was calculated for each shot taken by every volunteer (summarized in Supplementary Table S2), and the mean BrIC values were determined for each rifle (Figure 7). It can be noted that Volunteers 1 and 2 had higher BrIC values while firing rifle C, followed by rifle A and then B. However, Volunteer 3 had higher BrIC values while firing rifle A compared to rifles B and C. Rifle B exhibited the lowest BrIC values for only Volunteers 1 and 2. A one-way ANOVA analysis was conducted with a significance level of *α* = 0.05 to further analyze the differences between the rifles for each volunteer. A significant difference was observed between rifles in BrIC values for Volunteers 2 and 3, with *p*-values of 0.0377 and 0.0224, respectively. However, no significant difference was detected between the rifles for Volunteer 1, with a *p*-value of 0.0578.

Hereafter, when comparing the FE outcomes for Volunteers 1, 2, and 3, the reference is to the accelerative inputs measured for each individual shot, applied to the GHBMC FE model.

### 3.4 MPS_95_ calculation by rifle

**FIGURE 8:**
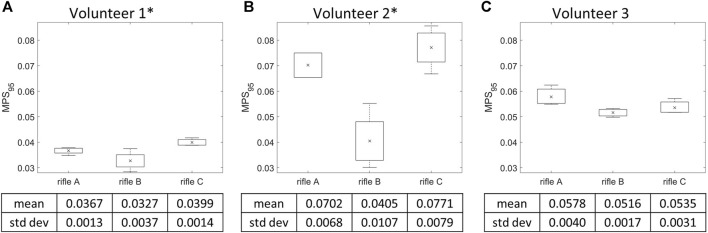
Mean MPS_95_ and variation of the three rifles for **(A)** Volunteer 1, **(B)** Volunteer 2, **(C)** Volunteer 3. * = statistically significant different detected between rifles. Number of trials for Volunteer 1 (rifles A:4, B:4, C:4), Volunteer 2 (rifles A:2, B:4, C:4) and Volunteer 3 (rifles A:3, B:3, C:3).

MPS_95_ for the whole brain was calculated for each simulation (summarized in Supplementary Table S3), and the mean MPS_95_ value was calculated for each rifle across all volunteers (Figure 8). Volunteers 1 and 2 had higher mean MPS_95_ values when firing rifle C followed by A then B. However, the mean MPS_95_ value of rifle A was slightly higher than rifle C for Volunteer 3. A one-way ANOVA analysis was conducted with a significance level of 0.05 to further analyze the differences between the rifles for each volunteer. A significant difference was observed between rifles for Volunteers 1 and 2, with *p*-values of 0.00741 and 0.001905, respectively. However, no statistically significant difference was detected between the rifles for Volunteer 3, with a *p*-value of 0.1139.

### 3.5 Cumulative strain-volume curves by rifle for the whole brain

**FIGURE 9:**
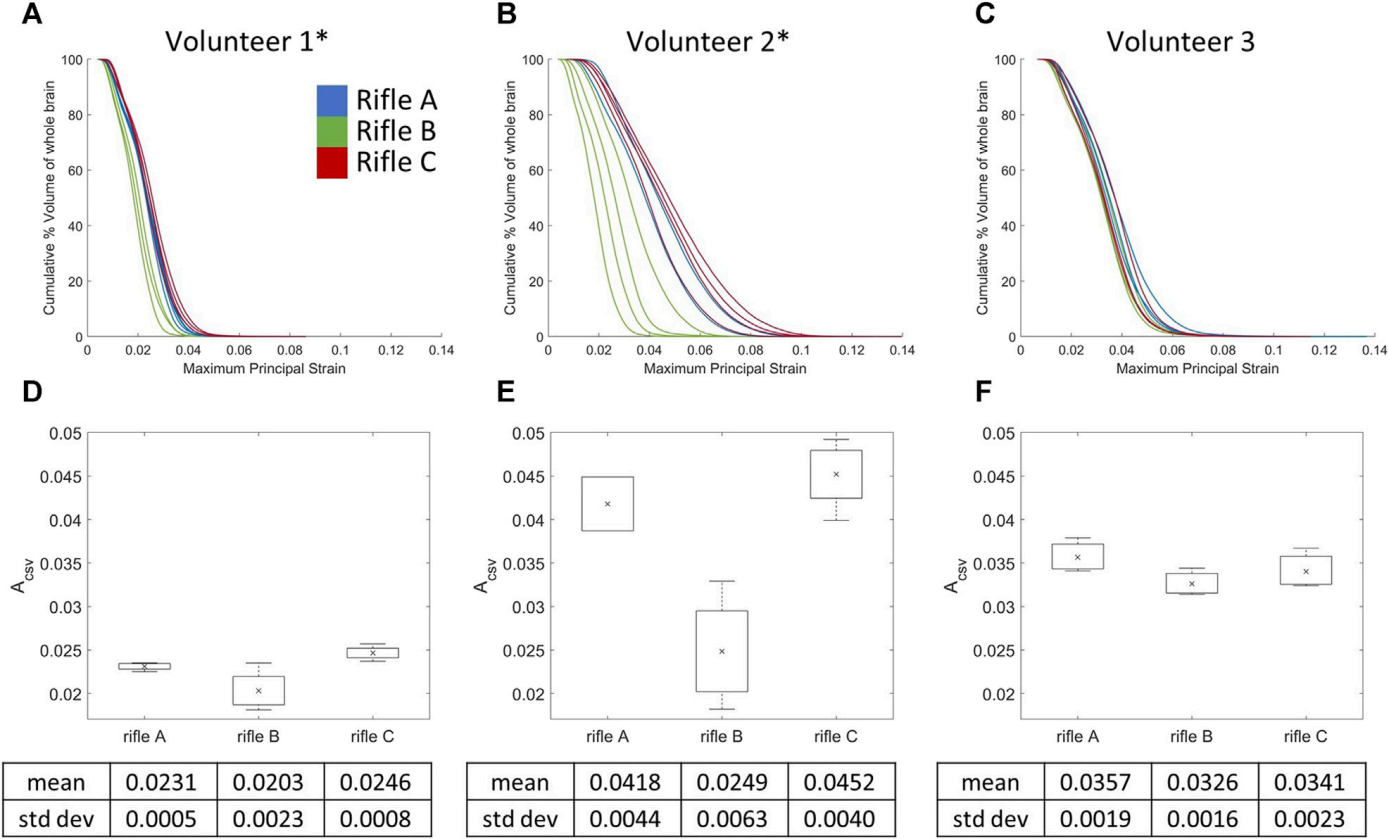
Cumulative strain-volume curves for each shot across the three rifles for **(A)** Volunteer 1, **(B)** Volunteer 2 and **(C)** Volunteer 3. Mean A_CSV_ variation for all three rifles of **(D)** Volunteer 1, **(E)** Volunteer 2 and **(F)** Volunteer 3. * = statistically significant different detected between rifles. Number of trials for Volunteer 1 (rifles A:4, B:4, C:4), Volunteer 2 (rifles A:2, B:4, C:4) and Volunteer 3 (rifles A:3, B:3, C:3).

The CSV curves of the whole brain were plotted for each shot taken by all volunteers (Figures 9A–C). The mean A_CSV_ (Figures 9D–F) was calculated for rifles A, B, and C for each volunteer (summarized in Supplementary Table S4). A one-way ANOVA analysis using A_CSV_ values was conducted with a significance level of 0.05 to further analyze the differences between the rifles for each volunteer. A significant difference was observed between rifles for Volunteers 1 and 2, with *p*-values 0.00691 and 0.00204, respectively. However, no statistically significant difference was detected between the rifles for Volunteer 3, with a *p*-value 0.242.

The cumulative strain-volume curves for Volunteers 1 and 2 revealed a decreasing severity of loading between the three rifles, although shape of the curve and the amount of variability per shot varied between volunteers (Figures 9A, B). In contrast, there was no observable difference in the cumulative strain-volume curves for Volunteer 3 across the three rifles (Figure 9C).

### 3.6 Cumulative strain-volume curves by volunteer for the whole brain

**FIGURE 10:**
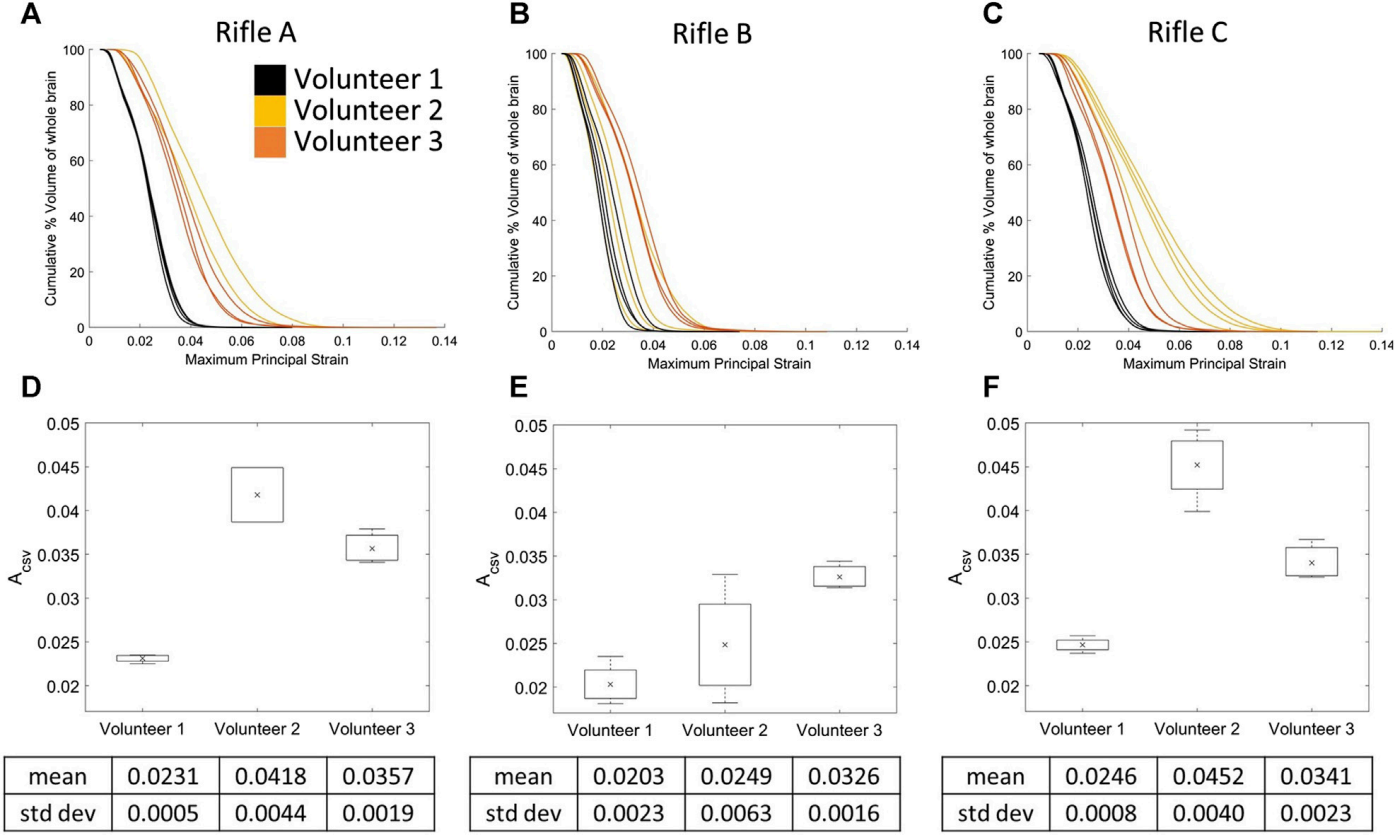
Cumulative strain plots for each shot taken by all three volunteers using rifle A **(A)**, rifle B **(B)**, and rifle C **(C)**. Mean A_CSV_ variation for all three volunteers using **(D)** rifle A, **(E)** rifle B and **(F)** rifle C. Number of trials for Volunteer 1 (rifles A:4, B:4, C:4), Volunteer 2 (rifles A:2, B:4, C:4) and Volunteer 3 (rifles A:3, B:3, C:3).

The CSV curves were plotted for each rifle, focusing on identifying trends between volunteers (Figures 10A–C). The mean A_CSV_ was calculated for Volunteers 1, 2, and 3 when firing each rifle (summarized in Supplementary Table S5). Volunteer 1 consistently experienced lower strains compared to the other two volunteers, irrespective of the rifle being fired, while Volunteer 2 clearly experienced higher strains when firing rifles, A and C. Another noteworthy aspect was consistency between shots of every volunteer for each rifle. Volunteer 1 and 3 had the least scatter between shots, regardless of the rifle. However, there was a noticeable increase in scatter for Volunteers 1 and 2 when firing rifle B compared to rifles A and C.

### 3.7 Response of individual brain regions

MPS_95_ values and CSV curves of each shot were extracted for the eight individual brain regions that make up the GHBMC M50 head model (summarized in Supplementary Section S3). The aim of this analysis was to investigate differences between brain regions per rifle and examine whether specific regions experienced higher strains.

The mean A_CSV_ and mean MPS_95_ was calculated for each of the 8 brain regions. A one-way ANOVA with a significance level of 0.05 was performed on both MPS_95_ and A_CSV_. There were significant differences between rifles for Volunteers 1 and 2 using both metrics. However, no significant differences were observed for Volunteer 3. The corresponding *p*-values for each rifle within each brain region are summarized in Supplementary Section S4. Across all eight brain regions, a consistent trend was observed in the CSV curves, with higher strains observed for rifle C, followed by rifle A and then rifle B.

**FIGURE 11:**
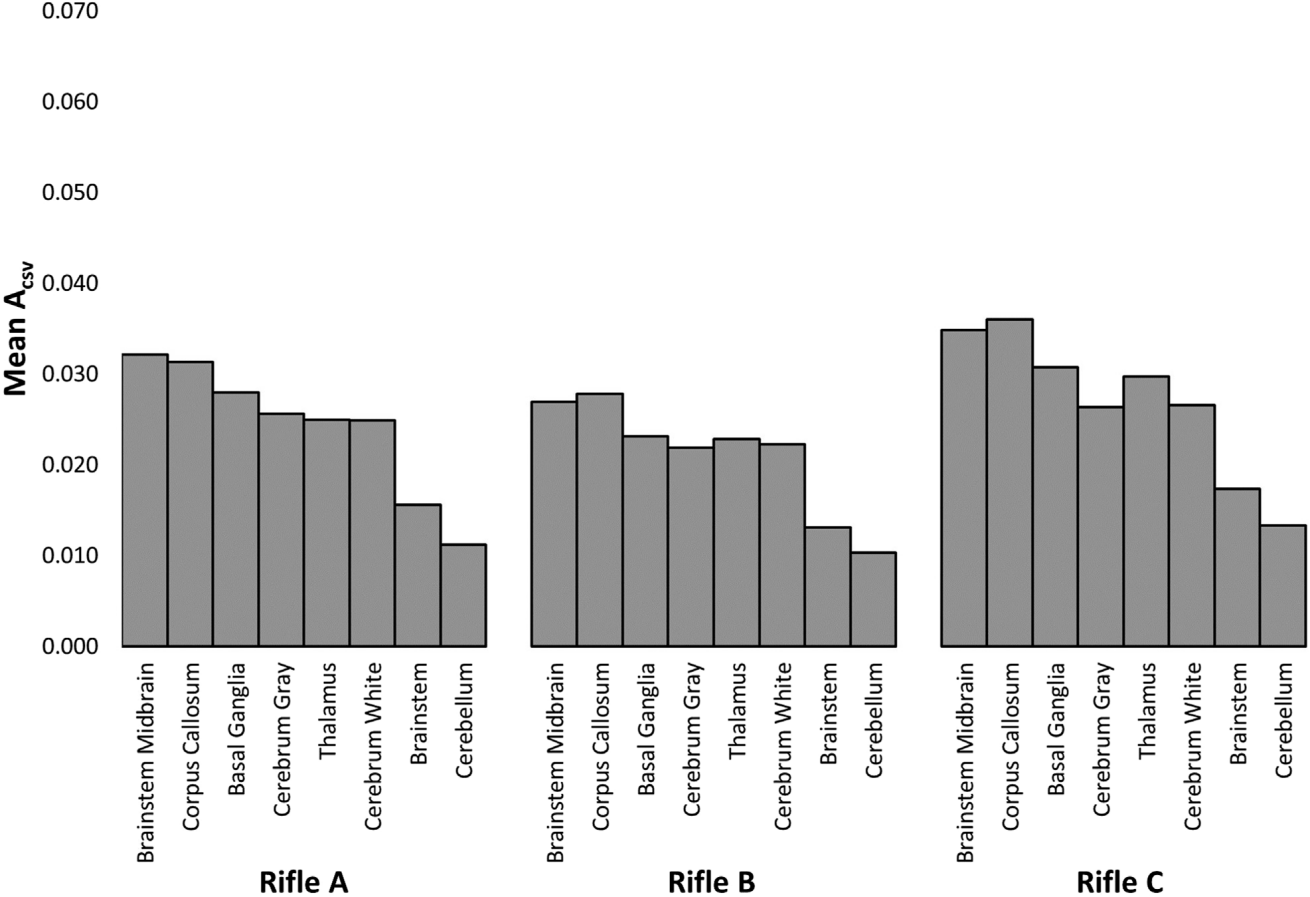
The area under the cumulative strain-volume curves (A_CSV_) was measured for each brain region while Volunteer 1 fired rifles A, B and C.

Figure 11 summarizes the A_CSV_ observed in each brain region for Volunteer 1 when firing rifles, A, B, and C (see Supplementary Figures S29, S30 for Volunteers 2 and 3). It can be observed that the brainstem midbrain and corpus callosum had the highest mean A_CSV_ for all cases considered across all three volunteers whereas the brainstem and cerebellum had the lowest mean A_CSV_. The order varied for the remaining regions when the same volunteer fired different rifles.

## 4 Discussion

### 4.1 Rifle comparison using measured head kinematics

In this study, the brain deformation of three CAF volunteers subjected to recoil forces when firing long range rifles was assessed and quantified using three metrics. This experimental set-up was unique as it enabled head kinematics measurements in a controlled environment, such as maintaining the same direction of impact between shots. Both rotational and translational motions in all three planes was recorded. As indicated by previous research (Hernandez et al., 2015), the combination of such kinematics may lead to increased injury severity.

Slight differences in kinematics (Figure 4; Supplementary Figures S1, S2) were observed between the three rifles used by each volunteer, indicating that different types of chassis resulted in varying input to the volunteers’ shoulders, despite each rifle using the same ammunition. Rifle B, which shared the same chassis as Rifle A but included the RMS, consistently exhibited lower measured head kinematics across all three volunteers, implying that the added RMS may have effectively reduced the kinematic impact on the volunteers’ heads. The data from Volunteers 1 (Figure 4) and 2 (Supplementary Figure S1) showed that rifle C produced higher kinematic values compared to rifle A. This suggests that the chassis type may be linked to increased recoil, or that the design of the chassis influenced the firing techniques of volunteers, resulting in higher head kinematics.

While differences in head kinematics were observed between rifle configurations, much larger distinctions can be observed between volunteers. For example, when examining the measured head kinematics for Volunteer 3, the visual differences between the rifles were less pronounced. This implies that Volunteer 3 may have a specific firing technique that minimized differences in head kinematics between rifle configurations. These comparisons suggest that when seeking to reduce head kinematics in volunteers firing long-range rifles, attention should be given not only to the chassis type and configuration but also to the unique firing techniques of each individual.

### 4.2 Rifle comparison using kinematics-based measures

Two kinematic-based metrics were calculated in this study: HIC_15_ and BrIC. The HIC_15_ and BrIC values observed in this study were several orders of magnitude lower in comparison to previously reported values in literature. For instance, a HIC_15_ value of 240 was reported for reversible brain injury in professional football players (Zhang et al., 2004), as well as HIC_15_ values of 22.5, 12.5, and 48.5 for football, hockey, and soccer, respectively, with no incidents of concussion (Naunheim et al., 2000). Similarly, BrIC values corresponding to a 50% probability of AIS 1 and 4 injuries, using the MPS-based injury risk curve reported by Takhounts et al. (2013) were approximately 0.15 and 1.1 respectively. Hence, the low HIC_15_ and BrIC values measured in this work suggest that the individual shots in this study were non-injurious. These results suggest that the cumulative effects of rifle recoil exposure may lead to similar concussion symptoms, reinforcing the critical need to implement measures to mitigate such occupational exposures.

Of the three rifles, the HIC_15_ values were consistently lowest for rifle B across all volunteers, indicating that the RMS reduced the magnitude of kinematics experienced by the volunteers, which aligns with the observations when comparing the measured head kinematics curves. For Volunteer 1, the mean HIC_15_ was highest when using rifle C, followed by rifles A and B, mirroring the trend seen in the measured kinematic peaks. However, for Volunteers 2 and 3, the mean HIC_15_ values were higher with rifle A compared to rifle C. This observation contradicts the measured head kinematics findings for Volunteer 2.

It should be noted that HIC_15_ may not be the most suitable metric for this study due to its sensitivity to high-frequency noise (Simms and Wood, 2009). In the present study, a single trial (Volunteer 2 shot 1 Rifle A) had a short-duration high peak measurement in the head kinematic (Supplementary Figures S1A–C). This introduced bias into the HIC_15_ calculations, leading to higher mean HIC_15_ values for rifle A compared to rifle C for Volunteer 2. This observation underscores the notion that HIC_15_ values can be influenced by short-duration high peaks in the signals that may not be representative of gross kinematics and may produce values greater than the loading condition investigated.

Another limitation of HIC_15_ is that it only accounts for linear acceleration and ignores the influence of head rotation. Consequently, it may be unsuitable for assessing risk of mTBI by itself, which is primarily a shear strain injury (Holbourn, 1943). As such, BrIC, a rotational based metric was calculated for each rifle configuration used by every volunteer. The order in BrIC values between rifles mirrored the measured head kinematics observation for Volunteers 1 and 2 (rifle C exhibited the highest mean BrIC values, while rifle A and B displayed lower mean BrIC values). However, Volunteer 3 displayed a different trend between rifles compared to the other two volunteers. For Volunteer 3, rifle C had the lowest BrIC value, followed by rifle B and A. This trend in BrIC values for Volunteer 3 diverged from the trend observed when using HIC_15_ for the same volunteer. Consequently, BrIC suggests that the RMS system did not sufficiently reduce the kinematic input to Volunteer 3’s head to the extent that it was lower than that of both rifles A and C. BrIC also has some drawbacks as it considers only angular velocity. Additionally, metrics solely based on kinematics do not provide insights into region-specific responses of the brain (Meaney et al., 2014), which is why more recent studies often consider strain-based metrics.

### 4.3 Rifle comparison using strain-based metrics

Two brain deformation-based metrics were used in this study: MPS_95_ and CSV curves. Higher MPS_95_ values were observed for rifle C compared to rifles B and A for Volunteers 1 and 2, aligning with the kinematic trend. However, the MPS_95_ for rifle A was higher than that of rifle C for Volunteer 3, mirroring the observations when using HIC_15_. But unlike the BrIC observations for the same volunteer, rifle B had the lowest magnitude of MPS_95_.

It is worth noting that, similar to HIC_15_, the MPS_95_ values did not fall within the range of 0.06–0.448, considered injurious in literature (Rycman et al., 2023). Although MPS_95_ has been widely used to assess brain response (Gabler et al., 2016), this metric did not capture critical information about how deformation distribution across the brain, such as if brain deformation was focal or widely distributed.

In contrast to the single-value MPS_95_ metric, CSV curves provided a more comprehensive representation of the strain distribution within each brain region with respect to its volume and enabled greater qualification of differences between rifle configuration and volunteers. Volunteer 1 exhibited a clear distinction between the three rifles with higher magnitudes observed when firing rifle C, followed by rifle A, and rifle B (Figure 9A). Additionally, Volunteer 1 exhibited little variability between shots fired with the same rifle. For Volunteer 2, a similar trend was observed between the rifles; however, there was higher variability between shots fired with each rifle, particularly with rifle B (Figure 9B). On the other hand, there was a high degree of overlap between all the curves for Volunteer 3 (Figure 9C). This suggested that the strain experienced by a volunteer was highly dependent on the individuals themselves, rather than solely on the rifle.

The variations in MPS observed between volunteers firing a specific rifle (Figure 10) may be attributed to differences in their anthropometric factors. Several studies have shed light on the critical role of neck strength and muscle activation in mitigating the risk of sports-related concussions (SRC). Viano et al. (2007) theorized that a larger and stronger neck could reduce the impact of external forces acting on the head, thereby reducing the head’s kinematic response to impact and consequently lowering the likelihood of concussion. Building upon this concept, Eckner et al. (2018) conducted a pilot study focusing on the effect of targeted neck strengthening exercises in youth athletes, which led to increased neck strength and girth. Importantly, this increased neck strength was reported to lead to decreased head linear and angular velocity, suggesting that resistance training exercises have promise in reducing SRC risk among young athletes.

Another study (Tierney et al., 2005) delved into the timing and muscle activation patterns during external force application between males and females. It revealed that pre-activating neck muscles, particularly in males, resulted in 25% less angular acceleration during known force application trials compared to unknown trials. These findings, in addition to the work of Eckner et al. (2018), highlight the significance of neck strength and muscle activation in reducing the acceleration of the head during impacts, thereby offering a potential avenue for SRC prevention. Future studies should investigate such training for the sniper population, where head-neck resistance training to increase muscle strength and neck girth and activation of primary stabilizing muscles in anticipation of the recoil force may enhance their ability to absorb external forces and utilize dynamic stabilizers for protection against head injuries.

It is also important to note that consistent shots (i.e., low variability in response to recoil force) did not necessarily indicate a lower risk of injury. For instance, when examining the strain ranges for each volunteer in the CSV curves (Figures 9A–C), at 50% volume of the whole brain, it was observed that Volunteer 3 experienced higher strains. In contrast, Volunteer 1 exhibited higher variability in strain values but slightly lower in magnitudes compared to Volunteer 3. As such, the RMS helped reduce the recoil impulse, but its effectiveness varied for each volunteer. Furthermore, there was an increase in shot variability for all volunteers when using rifle B, which shared the same chassis as rifle A but featured an additional RMS. This could be attributed to the subjects’ lack of experience with the RMS, which in turn affected their consistency between shots. These findings provide additional evidence that experience and familiarity play a crucial role in consistency between shots and influences the amount of strain observed by a volunteer.

Interestingly, Volunteer 1 exhibited less coronal rotation compared to the other two volunteers (Figure 5A), which is known to be a contributing factor to injury (Patton et al., 2013; Hernandez et al., 2015). This could potentially explain why Volunteer 1 consistently had the lowest strain magnitudes compared to Volunteers 2 and 3 (Figure 10). In the current study, the CSV curve was used to provide information on the magnitude of deformation, and distribution of deformation in a given brain region. Future studies will investigate the possibility of using CSV to assess injury risk.

### 4.4 Response of individual brain regions

It was observed that the order of decreasing strain for the brain regions changed when the same volunteers used different rifles, and similarly, when different volunteers used the same rifle, different regions showed higher strain. However, in most cases, the corpus callosum was the region with the highest strain. A previous study suggested a possible explanation that large coronal rotation may generate a wave down the flax celebri, causing stress in the corpus callosum (Hernandez et al., 2015). As the primary function of the corpus callosum is to transmit information between cerebral hemispheres, damage to this region can therefore disrupt communication, lead to disorientation, impaired vision, and other symptoms typical of mTBI. Additionally, the midbrain, basal ganglia, and thalamus were identified as three other regions experiencing high strains, aligning with outcomes of previous studies (Viano et al., 2005; Patton et al., 2013; Hernandez et al., 2015; Patton et al., 2015). Understanding which regions and kinematic directions are more likely to cause injury is crucial. For example, trends in this study suggests that the design of protective equipment should incorporate mitigation of rotation about the coronal plane to potentially reduce strains in this population.

### 4.5 Limitations

This study had several limitations. For instance, although the mouthguard effectively detected differences in kinematics when firing different rifles, the volunteer rested their cheek on the buttstock of the rifle, which potentially introduced high frequency acceleration in the mouthguard signals due to coupling with the rifle. The origin of these short duration high magnitude peaks should be investigated further since they can influence certain head injury metrics such as HIC_15_.

In this study, the assumption is made that the maxilla is rigidly attached to the skull, allowing the motion measured at the dentition by the mouthguard to be translated into the head center of gravity. It is important to note that the firing technique of all volunteers includes resting the cheek against the buttstock of the rifle. Consequently, the kinematics measured by the mouthguards represent not only head accelerations occurring from the shoulder to the neck and head but also loading due to the cheek resting on the buttstock. Although it is possible for loading to occur to the head directly via the buttstock, the motion of the rifle is parallel to the cheek and underlying hard tissues, which suggests that direct loading is not the prominent load path to generate gross head accelerations.

While this particular population offered a controlled environment for data collection, it is worth considering that their threshold for injury may potentially be elevated compared to the general population, owing to the rigorous intensity of their training regimen. The individual concussion histories and cognitive tests of this study cohort was not performed to assess the cumulative effects. Consequently, the investigation only provides insights into head kinematics for a single recoil impulse.

The FE model used in this study, despite being validated using cadaveric data, did not consider anatomical and physiological variations between specific volunteers. Additionally, different types of head models have been used to estimate injury thresholds, and it is important to note that the predicted strain for the same kinematic input may vary among different FE models (Ji et al., 2014).

Despite each group under comparison consisting of only 4 data points (shots), ANOVA analysis was conducted under the assumption of normal distribution. Given the small sample size, other non-parametric tests were not explored in this study. It should be emphasized that small dataset sizes are an inherent limitation in studies of this nature. This limitation is driven by the primary study objective, which aims to gain a comprehensive understanding of the injury mechanisms to ultimately minimize exposure risk. Striking this balance between comprehending injury mechanisms and minimizing risk naturally leads to the presence of relatively small datasets, thereby challenging the conduct of statistical analysis. However, the analysis did provide valuable insights into the observed trends.

## 5 Conclusion

The head kinematics of three volunteers from the CAF were experimentally measured using an instrumented mouthguard while firing three different rifles. The measured head kinematics were inputted to the GHBMC 50^th^ male head model to analyze the variations in brain strains during rifle firing. Four metrics based on head kinematics and brain deformation were investigated. It was found that the CSV curves were more sensitive and offered a better representation of the strain distribution in the brain than MPS_95_, BrIC and HIC_15_. This was because the CSV curves accounts not only for the degree of tissue deformation in the brain but also the degree to which the entire brain is loaded.

Differences between rifles and volunteers showed that rifle and training play a role in reducing the risk of injury. It was observed that when volunteers fired rifle C, higher strains were detected, whereas all volunteers exhibited lower strains when firing rifle B with the RMS, suggesting that the RMS effectively reduced recoil. Nevertheless, a noteworthy observation was the existence of a volunteer dependency, where Volunteer 1 consistently experienced lower strains in comparison to the other participants, irrespective of the rifle being fired. This observation indicated that not only protective equipment but also firing technique can influence the magnitude of strain induced during such events. Furthermore, certain brain regions, such as the corpus callosum and brainstem midbrain, consistently demonstrated higher strain levels compared to other regions, such as the cerebellum, which consistently exhibited lower strains. This observation suggested that specific regions may serve as more reliable indicators of mTBI.

This study provides foundational 0.50 caliber information on the magnitude of kinematics and brain deformation experienced by snipers when firing long-range rifles. While the measured kinematic and strain quantities in this population were much lower than those reported in the field of sports injury, many of the conclusions about the type and degree of loading, as well as specific regions of the brain susceptible to injury were observed in this work. The findings of this study provide an important first step to understanding, quantifying, and mitigating the risk of long-term chronic injury for persons exposed to repeated recoil forces.

## Data Availability

The original contributions presented in the study are included in the article/Supplementary Material, further inquiries can be directed to the corresponding author.
