# Supplementary material for: Assessment of brain response in operators subject to recoil force from firing long-range rifles
Source: Front Bioeng Biotechnol. 2024 Feb 14;12:1352387. doi: 10.3389/fbioe.2024.1352387 (PMC10899685; doi:10.3389/fbioe.2024.1352387)
Supplement: Supplementary file 1 [file DataSheet1.docx]

Supplementary Material

# Measured Head Kinematics for Volunteers 2 and 3

**
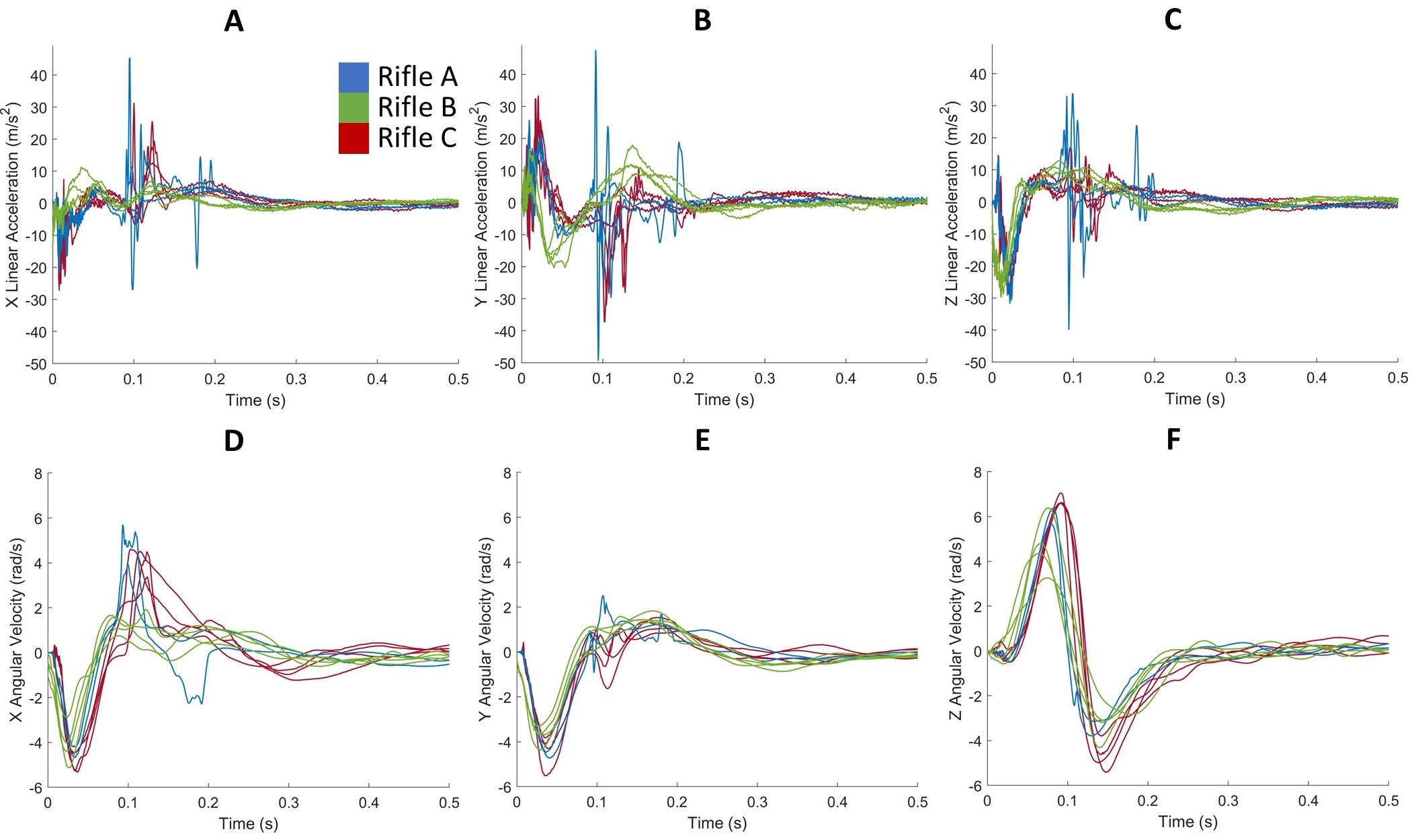
**

**Supplementary Figure 1. Exemplar case showing head kinematics ((A) linear acceleration in X, (B) linear acceleration in Y, (C) linear acceleration in Z, (D) angular velocity in X, (E) angular velocity in Y, (F) angular velocity in Z) for all shots taken by volunteer 2 using rifles A, B and C.**


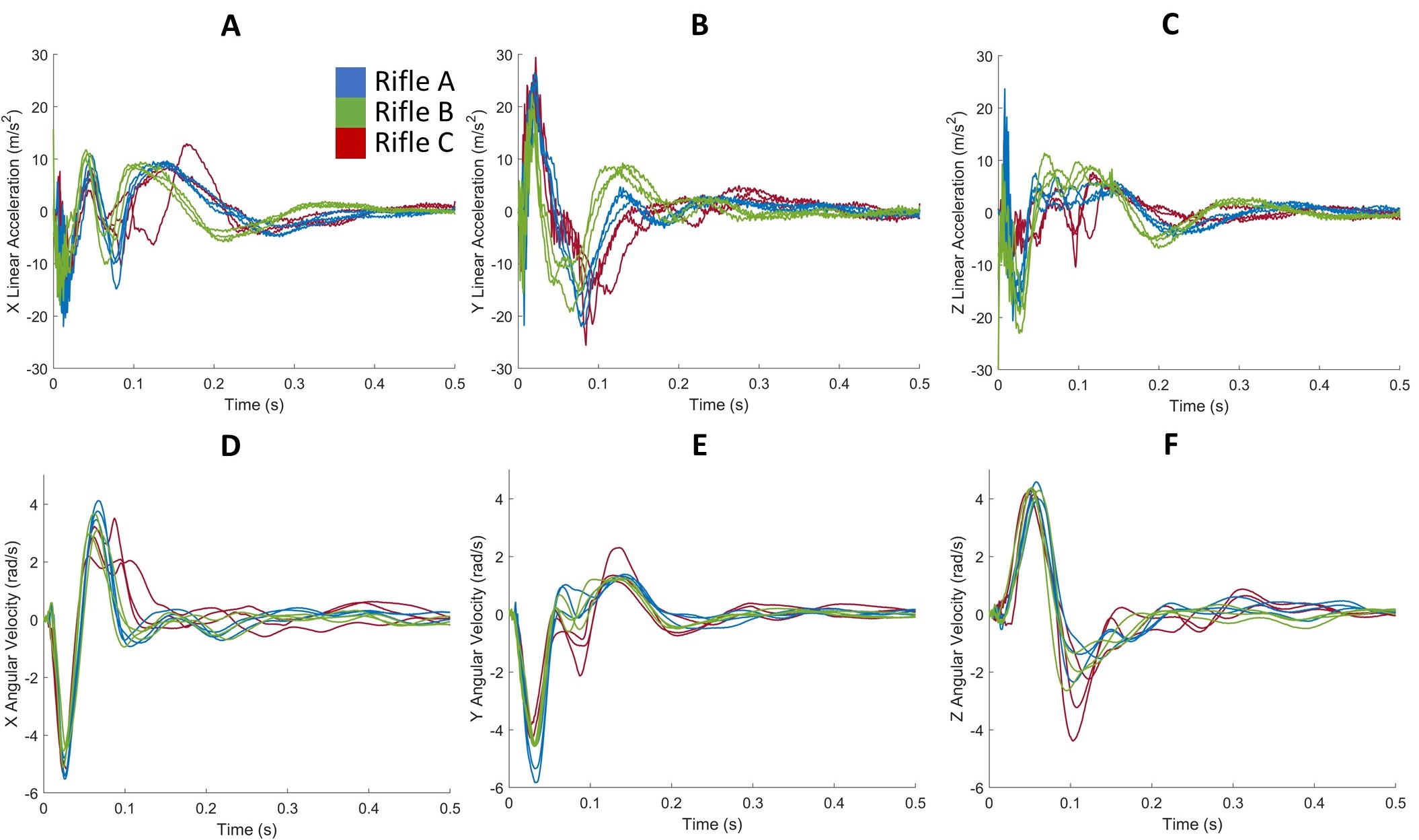


**Supplementary Figure 2.** **Exemplar case showing head kinematics ((A)linear acceleration in X, (B) linear acceleration in Y, (C) linear acceleration in Z, (D) angular velocity in X, (E) angular velocity in Y, (d) angular velocity in Z) for all shots taken by volunteer 3 using rifles A, B and C**.

**
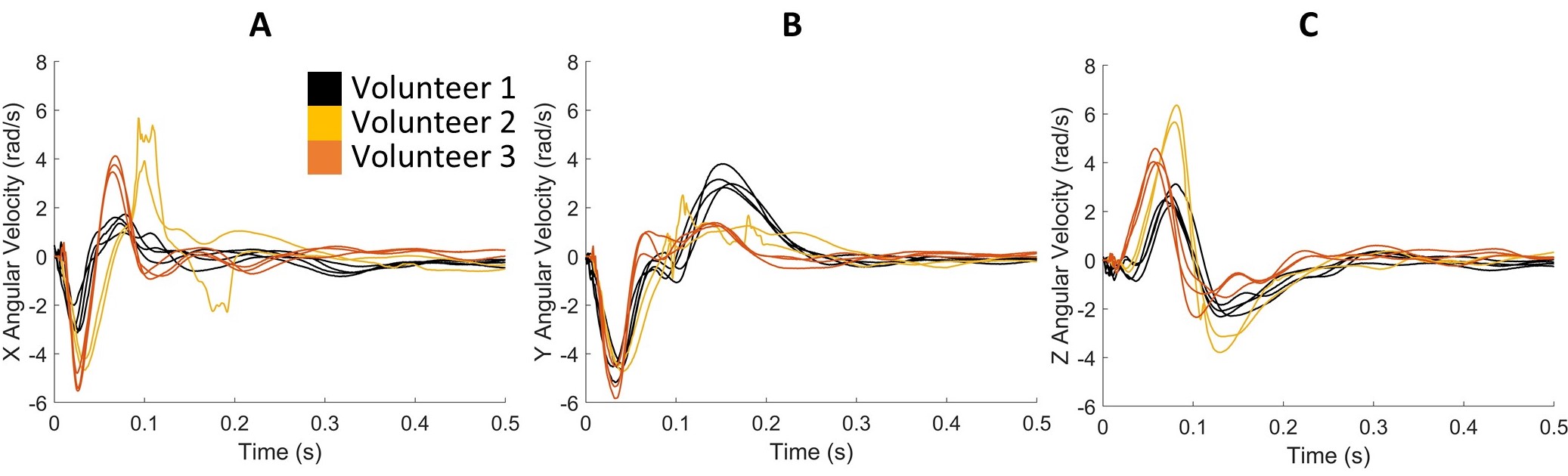
**

**Supplementary Figure 3.** **Exemplar case comparing the angular velocities (A) in X, (B) in Y and (C) in Z directions when volunteers are firing rifle A.**

**
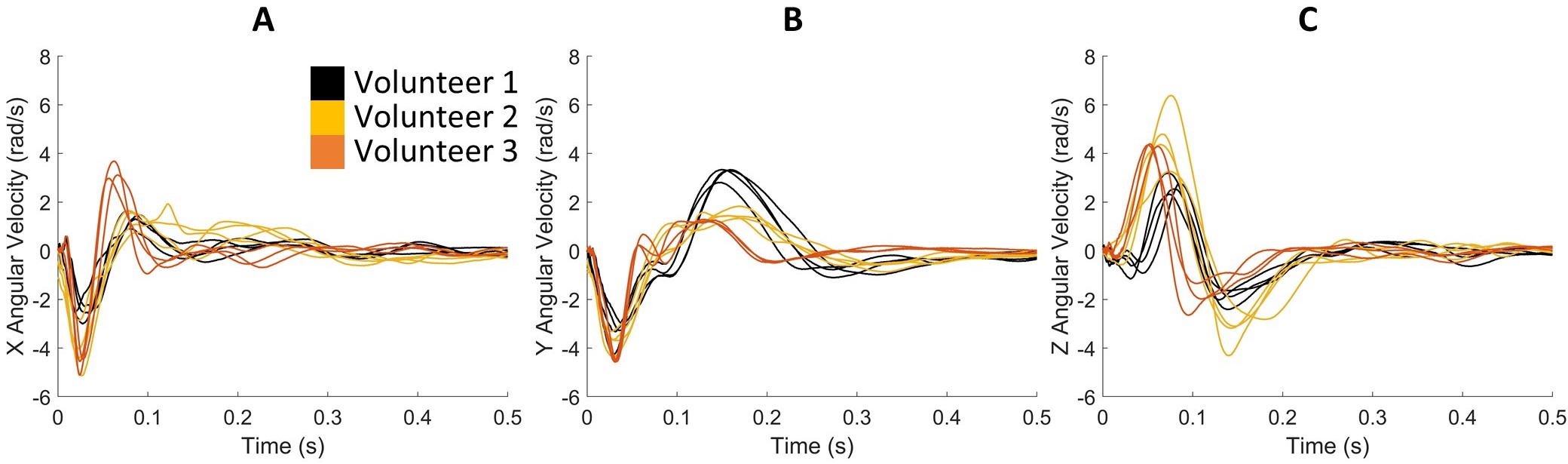
**

**Supplementary Figure 4.** **Exemplar case comparing the angular velocities (A) in X, (B) in Y and (C) in Z directions when volunteers are firing rifle B.**

# HIC_15_, BrIC, MPS_95_ and A_CSV_ for each shot of every volunteer

Supplementary Table 1: Summary of HIC_15_ values for volunteers 1,2,3 when firing rifles, A, B and C

|  | **Volunteer 1 rifle A** | **Volunteer 1 rifle B** | **Volunteer 1 rifle C** |
| --- | --- | --- | --- |
| shot 1 | 0.1402 | 0.07409 | 0.2205 |
| shot 2 | 0.1331 | 0.09631 | 0.2154 |
| shot 3 | 0.1593 | 0.08317 | 0.1823 |
| shot 4 | 0.1015 | 0.09091 | 0.1238 |
| mean HIC_15_ | **0.1335** | **0.0861** | **0.1855** |
| Sample standard deviation* | **0.0240** | **0.0097** | **0.0445** |
|  | **Volunteer 2 rifle A** | **Volunteer 2 rifle B** | **Volunteer 2 rifle C** |
| shot 1 | 0.3719 | 0.1109 | 0.2136 |
| shot 2 | 0.1456 | 0.1246 | 0.2645 |
| shot 3 | x | 0.1541 | 0.1733 |
| shot 4 | x | 0.1485 | 0.1889 |
| mean HIC_15_ | **0.2588** | **0.1345** | **0.2101** |
| Sample standard deviation | **0.1600** | **0.0203** | **0.0399** |
|  | **Volunteer 3 rifle A** | **Volunteer 3 rifle B** | **Volunteer 3 rifle C** |
| shot 1 | 0.165 | 0.1372 | 0.1338 |
| shot 2 | 0.203 | 0.06814 | 0.12 |
| shot 3 | 0.1615 | 0.09323 | 0.1326 |
| mean HIC_15_ | **0.1765** | **0.0995** | **0.1288** |
| Sample standard deviation | **0.0230** | **0.0350** | **0.0076** |

*Sample standard deviation = $\sqrt{\frac{\sum_{i}^{n} ({x_{i}-x_{mean})}^{2}}{n-1}}$

Where $x_{i}$ = observed values of a sample item, $x_{mean}$ = mean value of all observations, n = number of observations.

Supplementary Table 2: Summary of BrIC values for volunteers 1,2,3 when firing rifles, A, B and C

|  | **Volunteer 1 rifle A** | **Volunteer 1 rifle B** | **Volunteer 1 rifle C** |
| --- | --- | --- | --- |
| shot 1 | 0.1138 | 0.0940 | 0.1062 |
| shot 2 | 0.1233 | 0.0914 | 0.1170 |
| shot 3 | 0.1123 | 0.1192 | 0.1315 |
| shot 4 | 0.1153 | 0.0984 | 0.1225 |
| mean BrIC | **0.1162** | **0.1008** | **0.1193** |
| Sample standard deviation | **0.0049** | **0.0126** | **0.0106** |
|  | **Volunteer 2 rifle A** | **Volunteer 2 rifle B** | **Volunteer 2 rifle C** |
| shot 1 | 0.1815 | 0.1088 | 0.1887 |
| shot 2 | 0.1902 | 0.1395 | 0.1915 |
| shot 3 | x | 0.1860 | 0.2013 |
| shot 4 | x | 0.1550 | 0.2038 |
| mean BrIC | **0.1858** | **0.1474** | **0.1963** |
| Sample standard deviation | **0.0062** | **0.0321** | **0.0073** |
|  | **Volunteer 3 rifle A** | **Volunteer 3 rifle B** | **Volunteer 3 rifle C** |
| shot 1 | 0.1608 | 0.1561 | 0.1516 |
| shot 2 | 0.1673 | 0.1497 | 0.1500 |
| shot 3 | 0.1568 | 0.1510 | 0.1511 |
| mean BrIC | **0.1616** | **0.1523** | **0.1509** |
| Sample standard deviation | **0.0053** | **0.0034** | **0.0008** |

Supplementary Table 3: Summary of MPS_95_ values for volunteers 1,2,3 when firing rifles, A, B and C

|  | **Volunteer 1 rifle A** | **Volunteer 1 rifle B** | **Volunteer 1 rifle C** |
| --- | --- | --- | --- |
| shot 1 | 0.0348 | 0.0322 | 0.0388 |
| shot 2 | 0.0375 | 0.0284 | 0.0389 |
| shot 3 | 0.0367 | 0.0375 | 0.0417 |
| shot 4 | 0.0378 | 0.0327 | 0.0404 |
| mean MPS_95_ | **0.0367** | **0.0327** | **0.0399** |
| Sample standard deviation | **0.0013** | **0.0037** | **0.0014** |
|  | **Volunteer 2 rifle A** | **Volunteer 2 rifle B** | **Volunteer 2 rifle C** |
| shot 1 | 0.0750 | 0.0301 | 0.0856 |
| shot 2 | 0.0654 | 0.0357 | 0.0761 |
| shot 3 | x | 0.0552 | 0.0668 |
| shot 4 | x | 0.0409 | 0.0800 |
| mean MPS_95_ | **0.0702** | **0.0405** | **0.0771** |
| Sample standard deviation | **0.0068** | **0.0107** | **0.0079** |
|  | **Volunteer 3 rifle A** | **Volunteer 3 rifle B** | **Volunteer 3 rifle C** |
| shot 1 | 0.0549 | 0.0532 | 0.0518 |
| shot 2 | 0.0624 | 0.0498 | 0.0517 |
| shot 3 | 0.0561 | 0.0518 | 0.0571 |
| mean MPS_95_ | **0.0578** | **0.0516** | **0.0535** |
| Sample standard deviation | **0.0040** | **0.0017** | **0.0031** |

Supplementary Table 4: Summary of area under cumulative strain-volume curves (A_CSV_) for volunteers 1,2,3 when firing rifles, A, B and C (grouped by volunteers)

|  | **Volunteer 1 rifle A** | **Volunteer 1 rifle B** | **Volunteer 1 rifle C** |
| --- | --- | --- | --- |
| shot 1 | 0.0225 | 0.0193 | 0.0237 |
| shot 2 | 0.0235 | 0.0181 | 0.0245 |
| shot 3 | 0.0231 | 0.0235 | 0.0257 |
| shot 4 | 0.0234 | 0.0204 | 0.0247 |
| mean A_CSV_ | **0.0231** | **0.0203** | **0.0246** |
| Sample standard deviation | **0.0005** | **0.0023** | **0.0008** |
|  | **Volunteer 2 rifle A** | **Volunteer 2 rifle B** | **Volunteer 2 rifle C** |
| shot 1 | 0.0449 | 0.0182 | 0.0492 |
| shot 2 | 0.0387 | 0.0222 | 0.0450 |
| shot 3 | x | 0.0329 | 0.0399 |
| shot 4 | x | 0.0261 | 0.0467 |
| mean A_CSV_ | **0.0418** | **0.0249** | **0.0452** |
| Sample standard deviation | **0.0044** | **0.0063** | **0.0040** |
|  | **Volunteer 3 rifle A** | **Volunteer 3 rifle B** | **Volunteer 3 rifle C** |
| shot 1 | 0.0350 | 0.0344 | 0.0324 |
| shot 2 | 0.0379 | 0.0314 | 0.0330 |
| shot 3 | 0.0341 | 0.0320 | 0.0367 |
| mean A_CSV_ | **0.0357** | **0.0326** | **0.0341** |
| Sample standard deviation | **0.0019** | **0.0016** | **0.0023** |

Supplementary Table 5: Summary of area under cumulative strain-volume curves (A_CSV_) for volunteers 1,2,3 when firing rifles, A, B and C (grouped by rifles)

|  | **Volunteer 1 rifle A** | **Volunteer 2 rifle A** | **Volunteer 3 rifle A** |
| --- | --- | --- | --- |
| shot 1 | 0.0225 | 0.0449 | 0.0350 |
| shot 2 | 0.0235 | 0.0387 | 0.0379 |
| shot 3 | 0.0231 | x | 0.0341 |
| shot 4 | 0.0234 | x | x |
| mean A_CSV_ | **0.0231** | **0.0418** | **0.0357** |
| Sample standard deviation | **0.0005** | **0.0044** | **0.0019** |
|  | **Volunteer 1 rifle B** | **Volunteer 2 rifle B** | **Volunteer 3 rifle B** |
| shot 1 | 0.0193 | 0.0182 | 0.0344 |
| shot 2 | 0.0181 | 0.0222 | 0.0314 |
| shot 3 | 0.0235 | 0.0329 | 0.0320 |
| shot 4 | 0.0204 | 0.0261 | x |
| mean A_CSV_ | **0.0203** | **0.0249** | **0.0326** |
| Sample standard deviation | **0.0023** | **0.0063** | **0.0016** |
|  | **Volunteer 1 rifle C** | **Volunteer 2 rifle C** | **Volunteer 3 rifle C** |
| shot 1 | 0.0237 | 0.0492 | 0.0324 |
| shot 2 | 0.0245 | 0.0450 | 0.0330 |
| shot 3 | 0.0257 | 0.0399 | 0.0367 |
| shot 4 | 0.0247 | 0.0467 | x |
| mean A_CSV_ | **0.0246** | **0.0452** | **0.0341** |
| Sample standard deviation | **0.0008** | **0.0040** | **0.0023** |

# MPS_95_ variation and cumulative strain-volume curves for the eight individual brain regions for each volunteer

## Volunteer 1


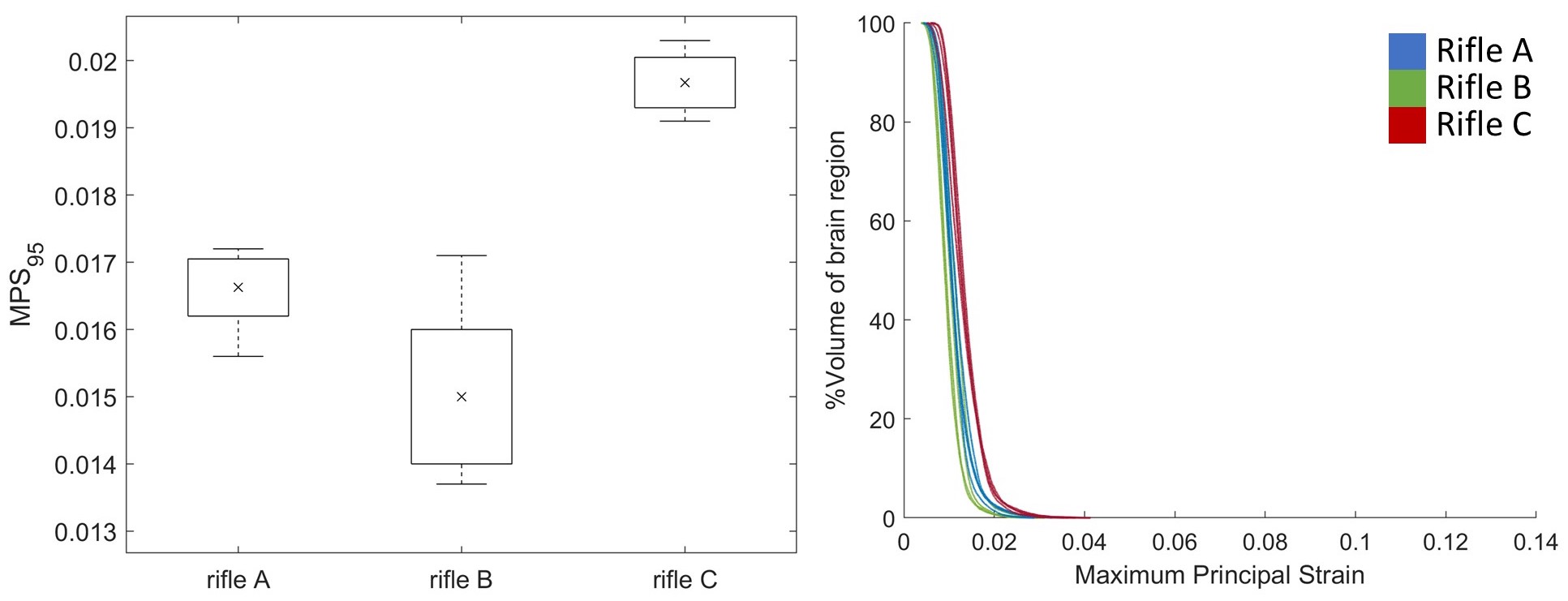


Supplementary Figure 5. Mean MPS_95_ and variation (A) and cumulative strain-volume curves (B) in the cerebellum across all three rifles for volunteer 1.


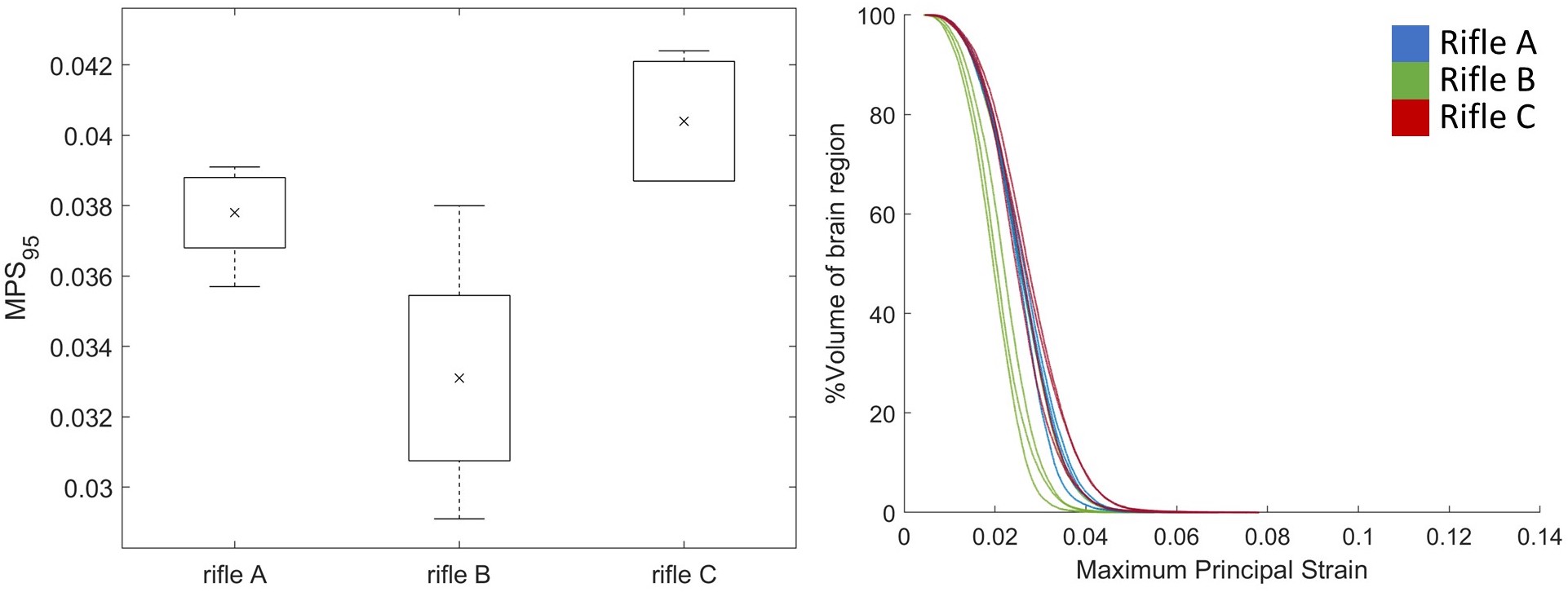


Supplementary Figure 6. Mean MPS_95_ and variation (A) and cumulative strain-volume curves (B) in the cerebrum gray region across all three rifles for volunteer 1.


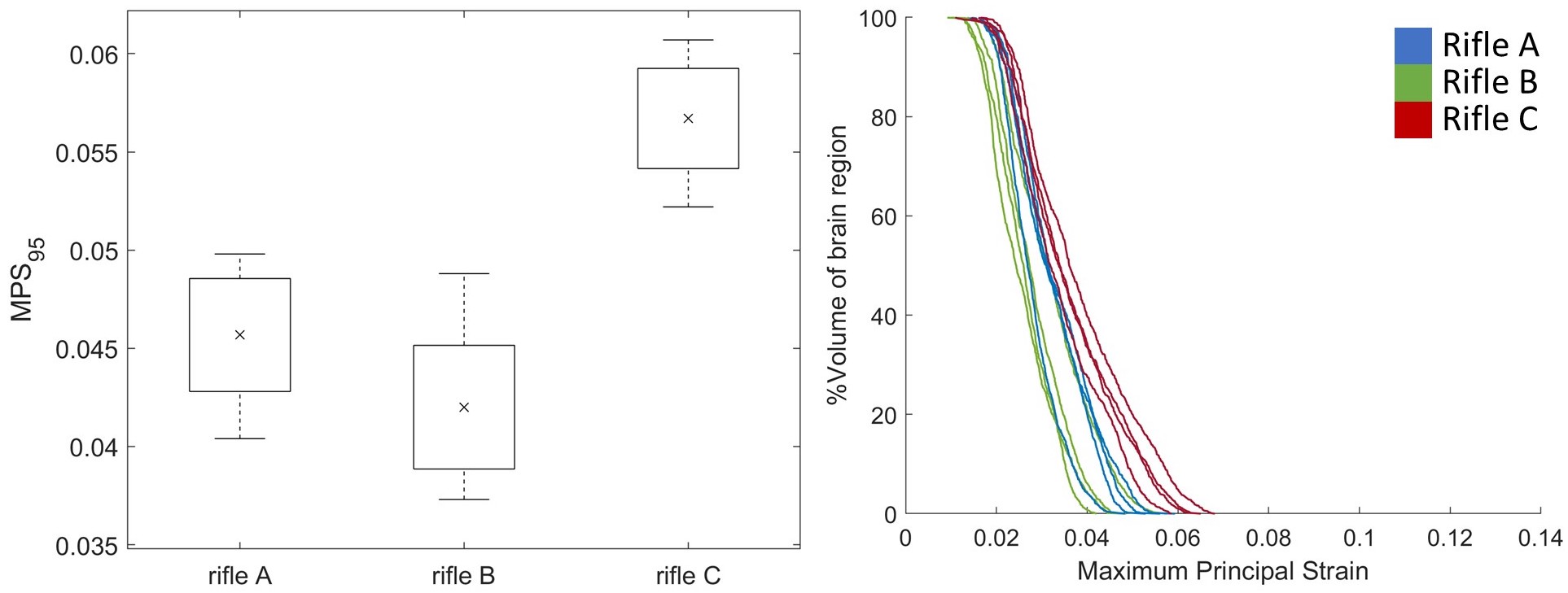


Supplementary Figure 7. Mean MPS_95_ and variation (A) and cumulative strain-volume curves (B) in the corpus callosum across all three rifles for volunteer 1.


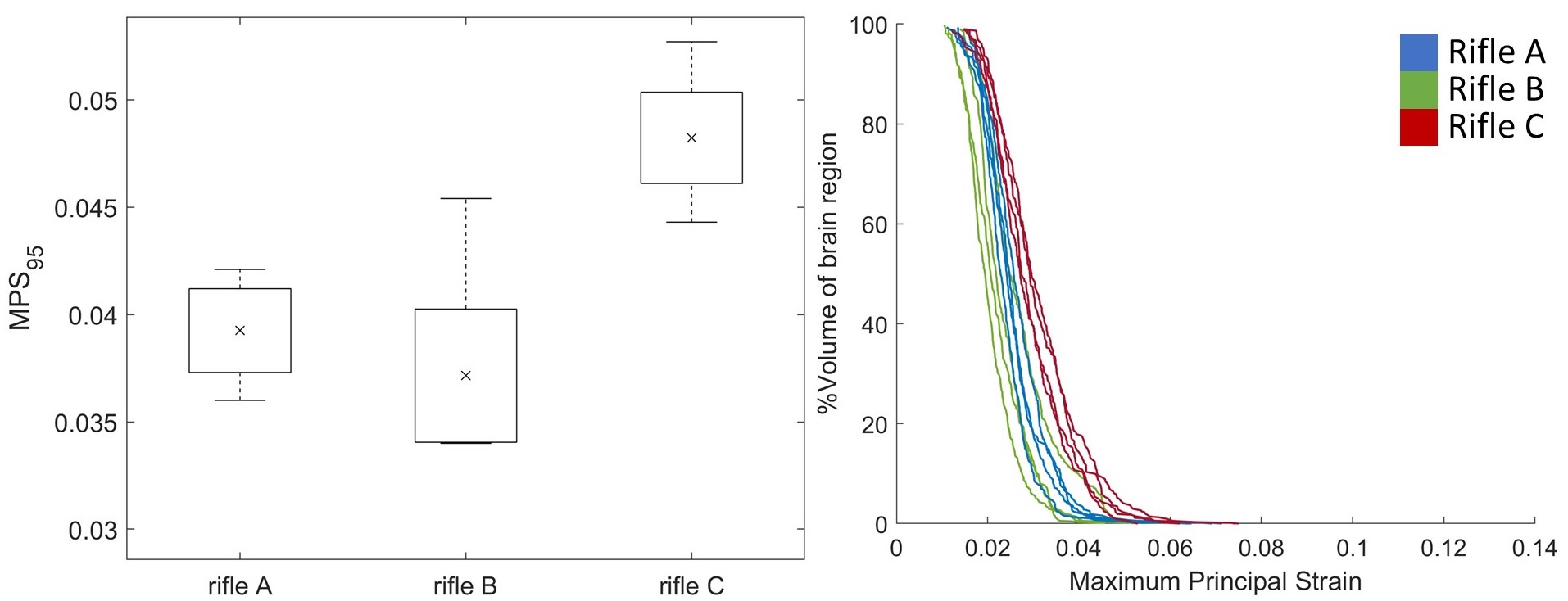


Supplementary Figure 8. Mean MPS_95_ and variation (A) and cumulative strain-volume curves (B) in the thalamus across all three rifles for volunteer 1.


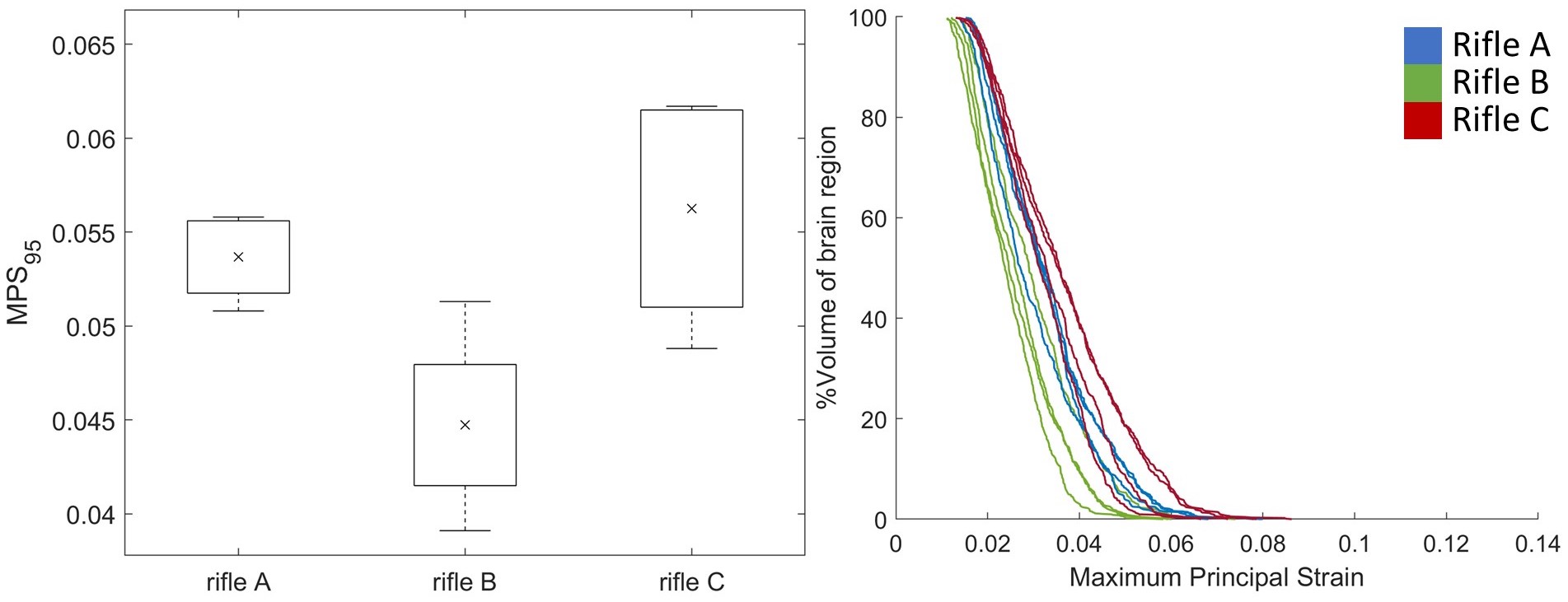


Supplementary Figure 9. Mean MPS_95_ and variation (A) and cumulative strain-volume curves (B) in the brainstem midbrain region across all three rifles for volunteer 1.


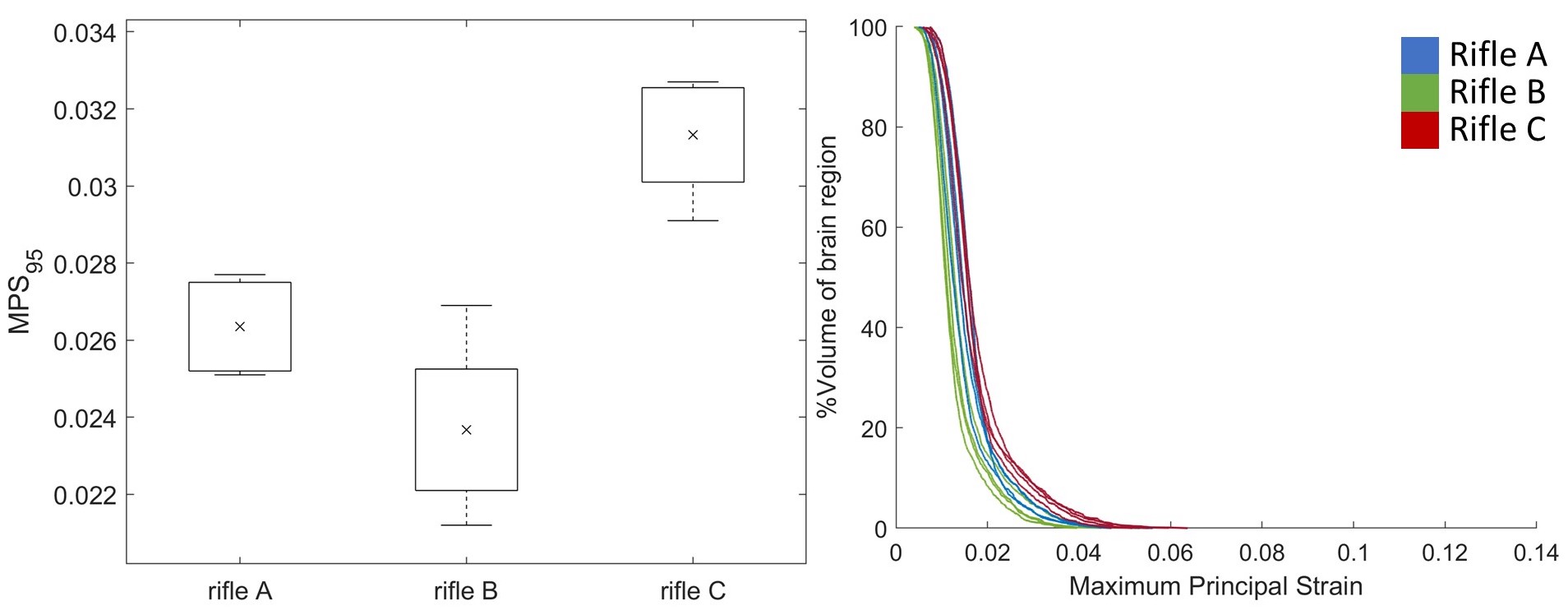


Supplementary Figure 10. Mean MPS_95_ and variation (A) and cumulative strain-volume curves (B) in the brainstem across all three rifles for volunteer 1.


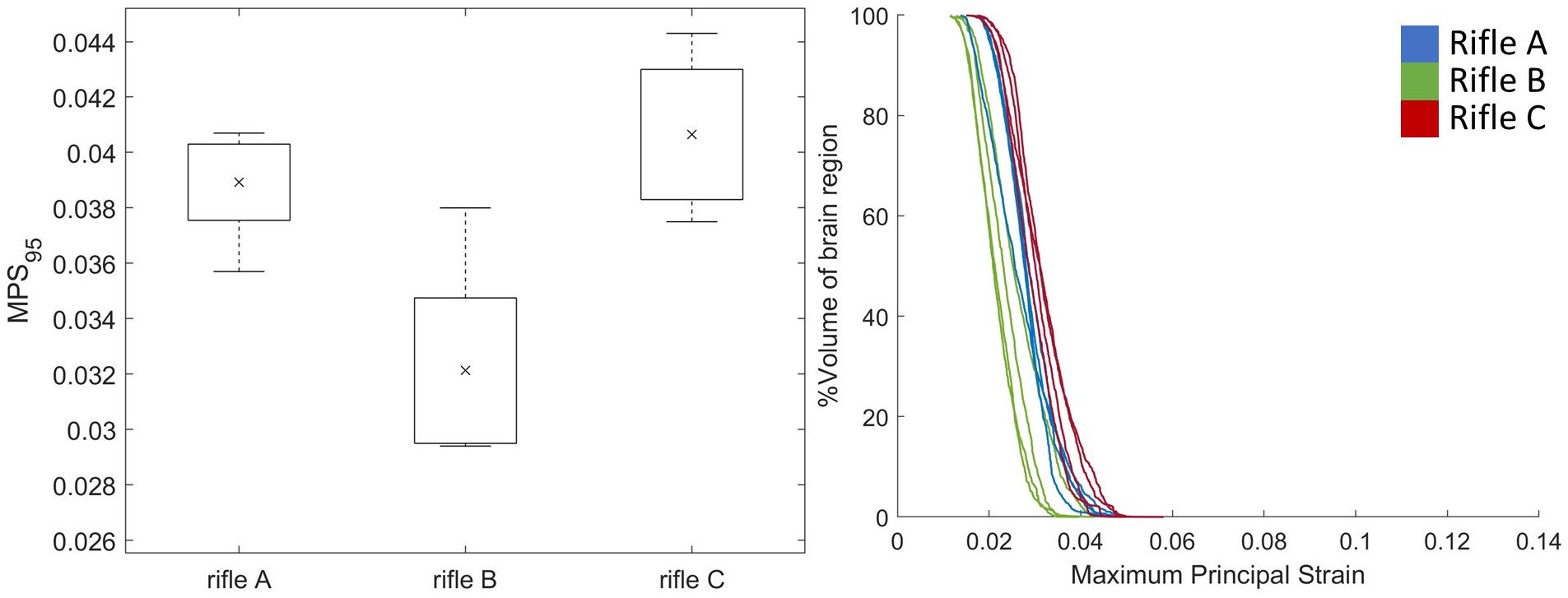


Supplementary Figure 11. Mean MPS_95_ and variation (A) and cumulative strain-volume curves (B) in the basal ganglia across all three rifles for volunteer 1.


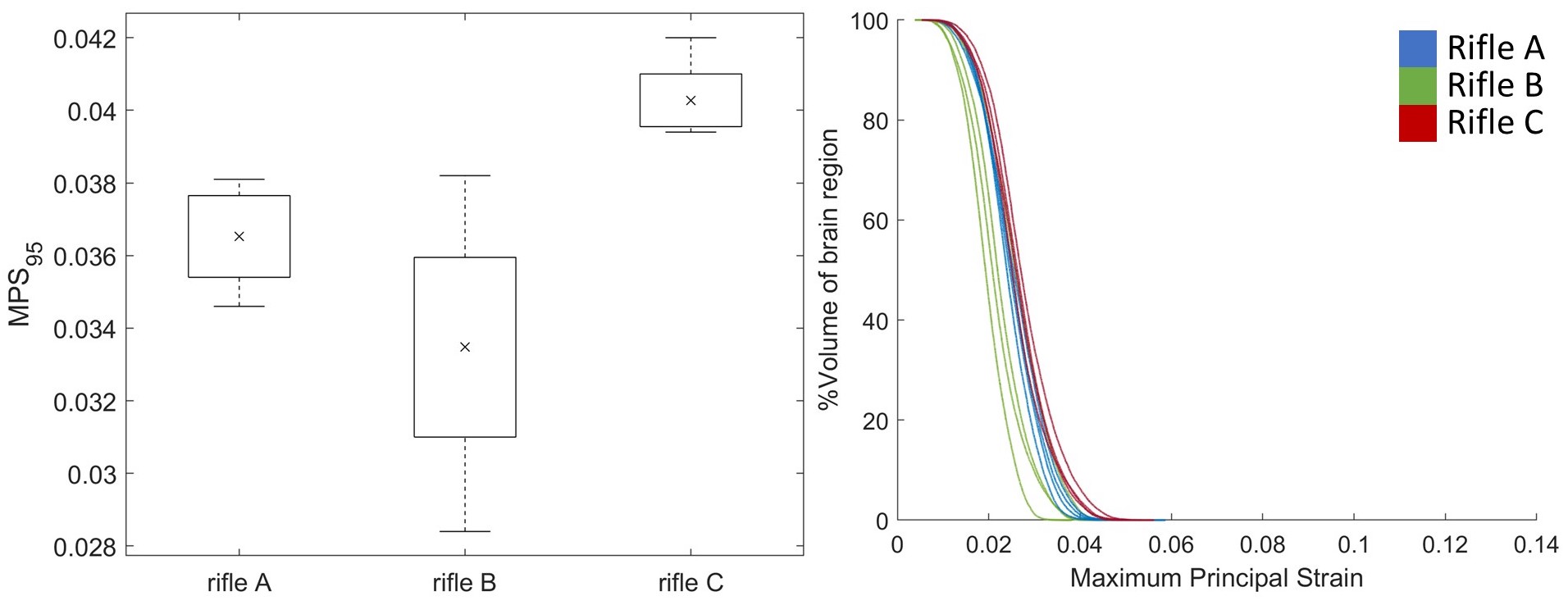


Supplementary Figure 12. Mean MPS_95_ and variation (A) and cumulative strain-volume curves (B) in the cerebrum white region across all three rifles for volunteer 1.

## Volunteer 2


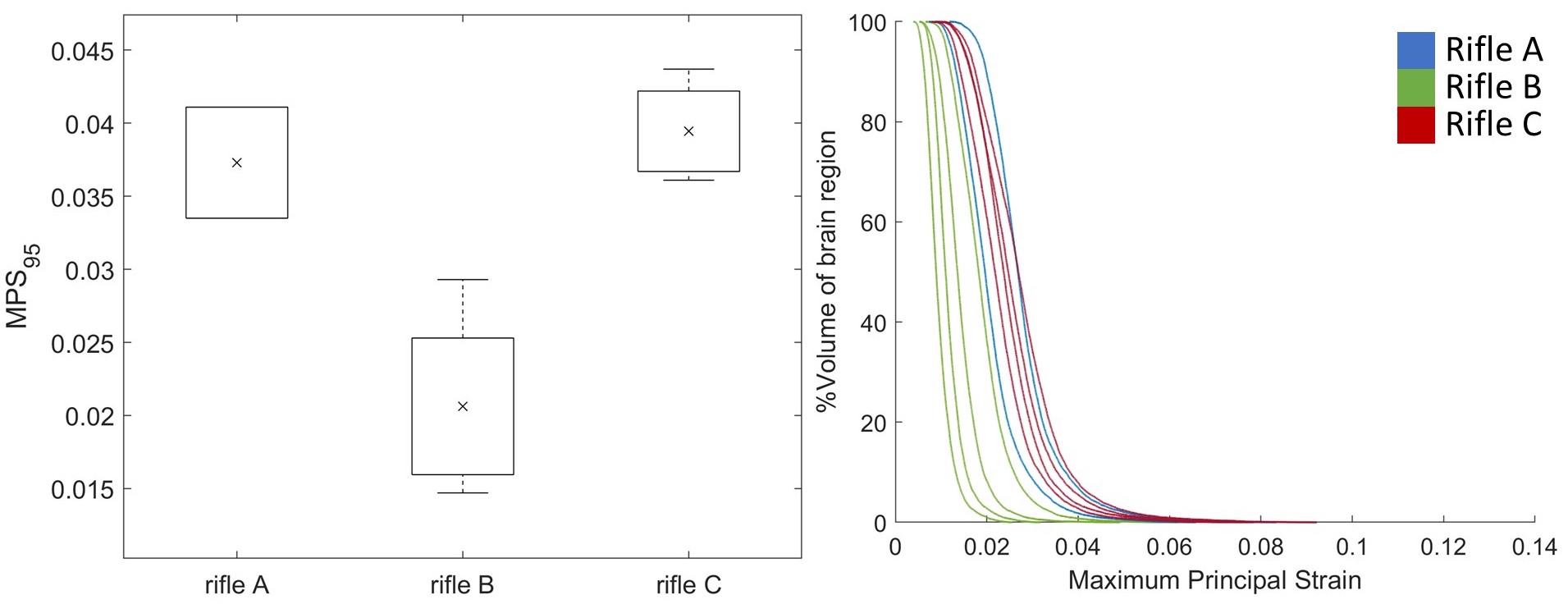


Supplementary Figure 13. Mean MPS_95_ and variation (A) and cumulative strain-volume curves (B) in the cerebellum across all three rifles for volunteer 2.


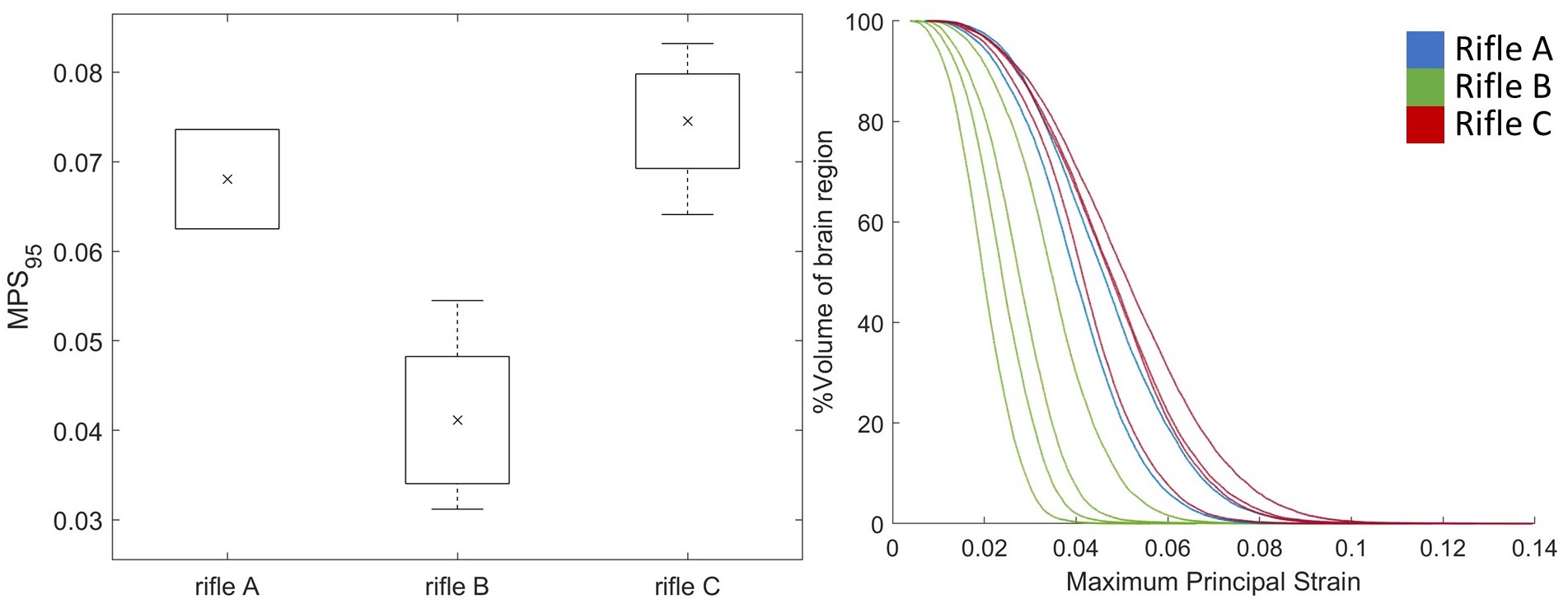


Supplementary Figure 14. Mean MPS_95_ and variation (A) and cumulative strain-volume curves (B) in the cerebrum gray region across all three rifles for volunteer 2.


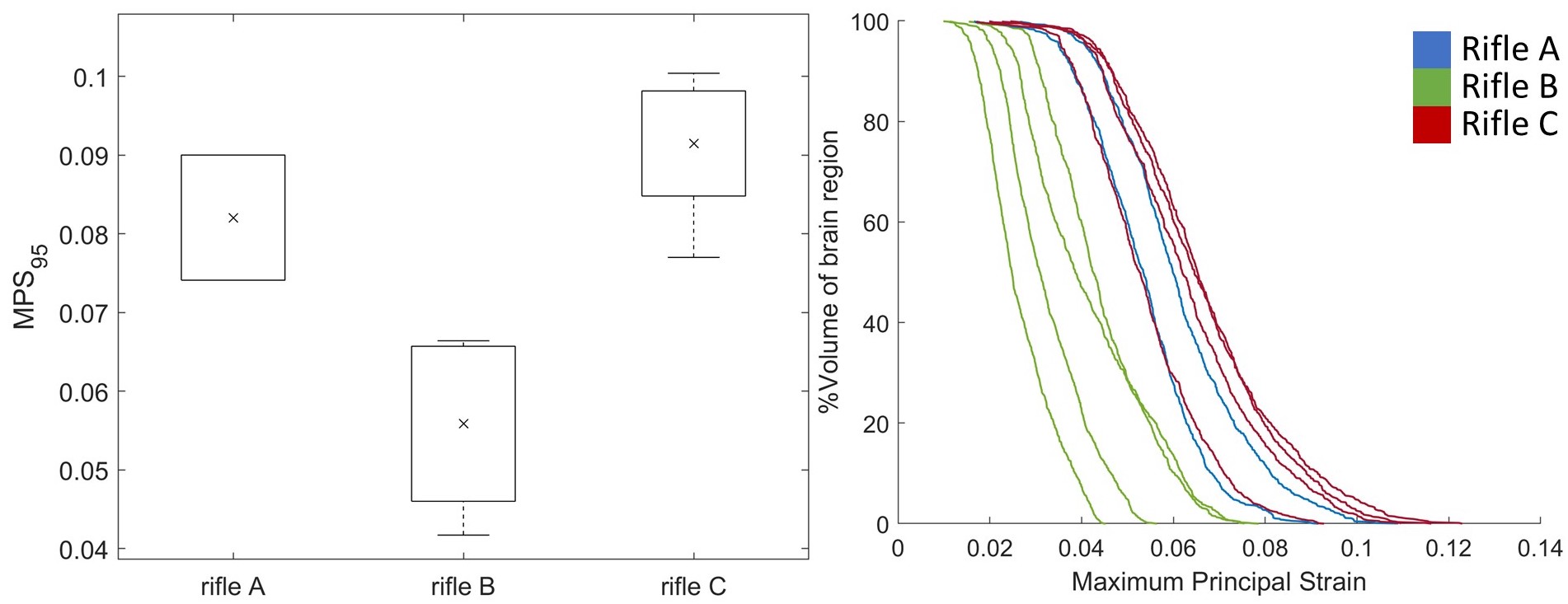


Supplementary Figure 15. Mean MPS_95_ and variation (A) and cumulative strain-volume curves (B) in the corpus callosum across all three rifles for volunteer 2.


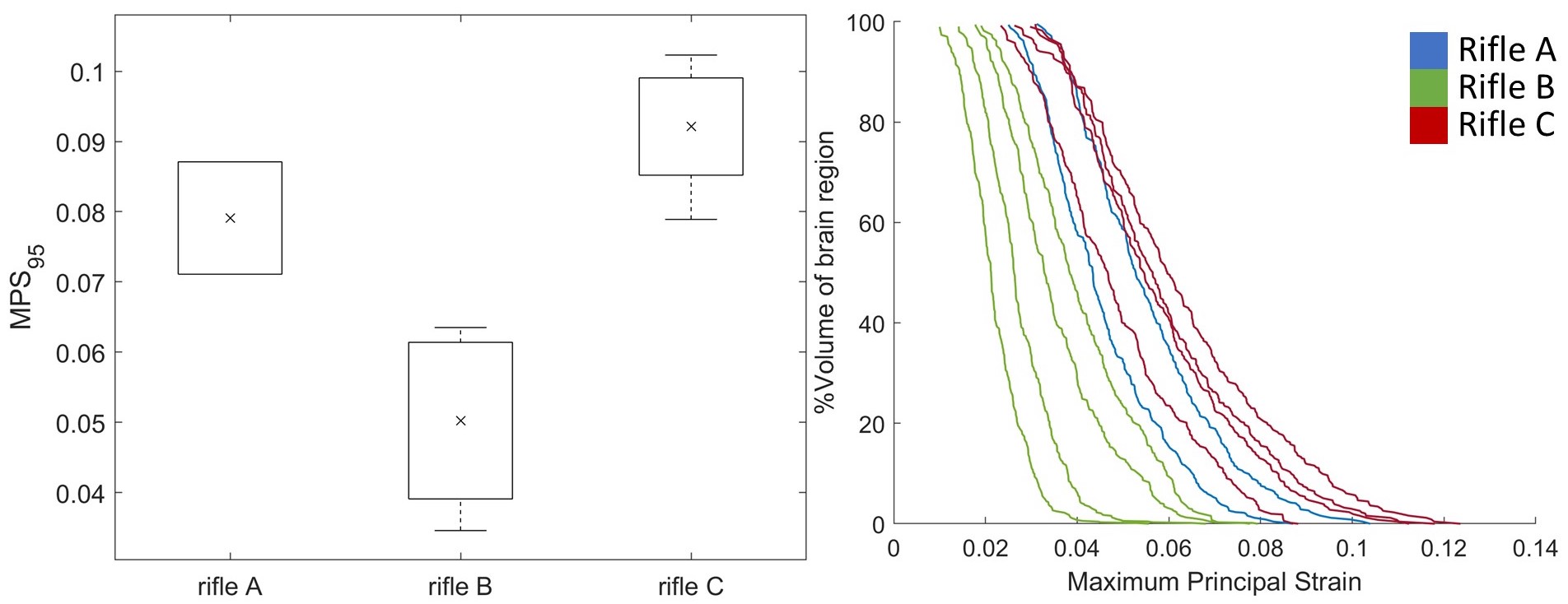


Supplementary Figure 16. Mean MPS_95_ and variation (A) and cumulative strain-volume curves (B) in the thalamus across all three rifles for volunteer 2.


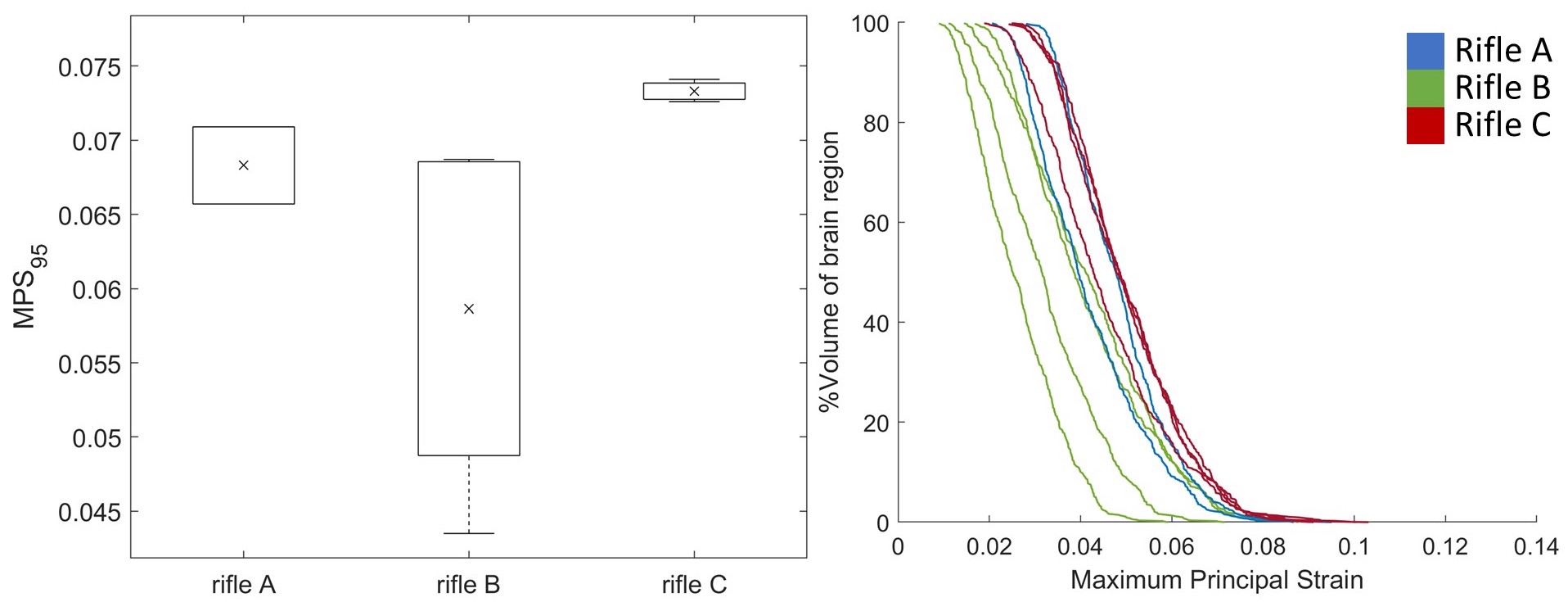


Supplementary Figure 17. Mean MPS_95_ and variation (A) and cumulative strain-volume curves (B) in the brainstem midbrain region across all three rifles for volunteer 2.


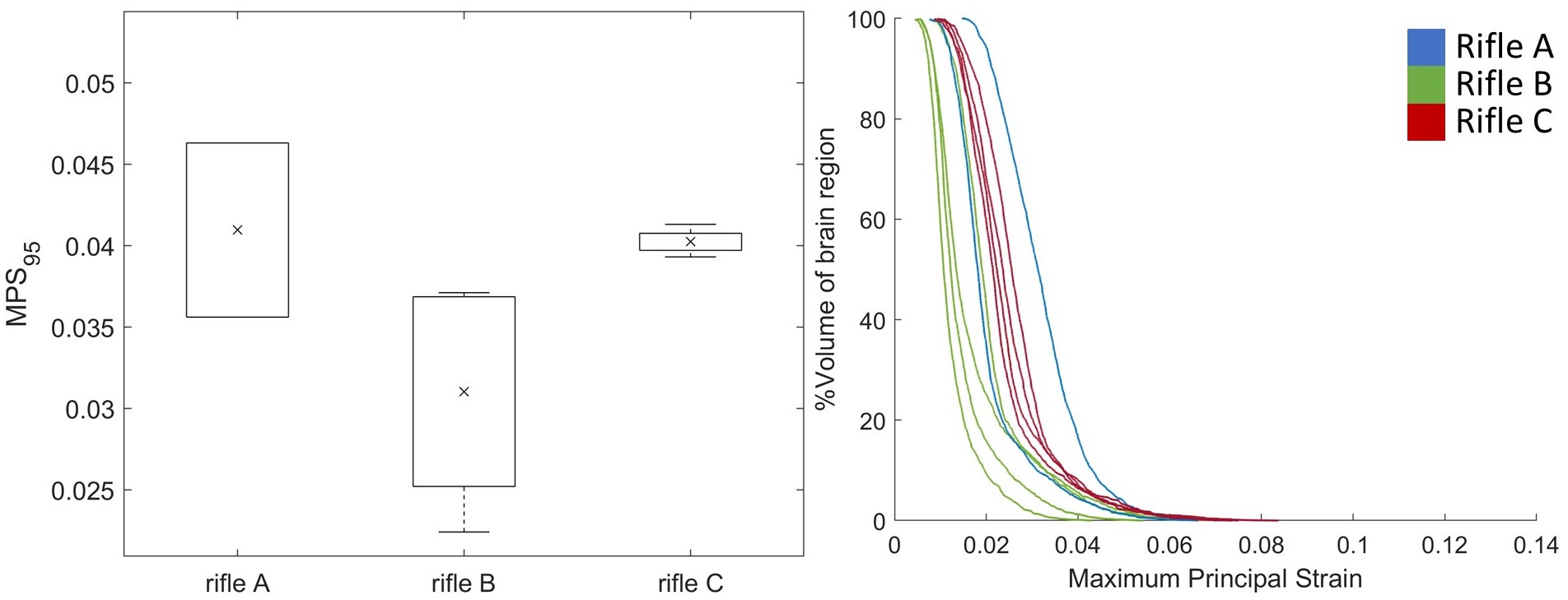


Supplementary Figure 18. Mean MPS_95_ and variation (A) and cumulative strain-volume curves (B) in the brainstem across all three rifles for volunteer 2.


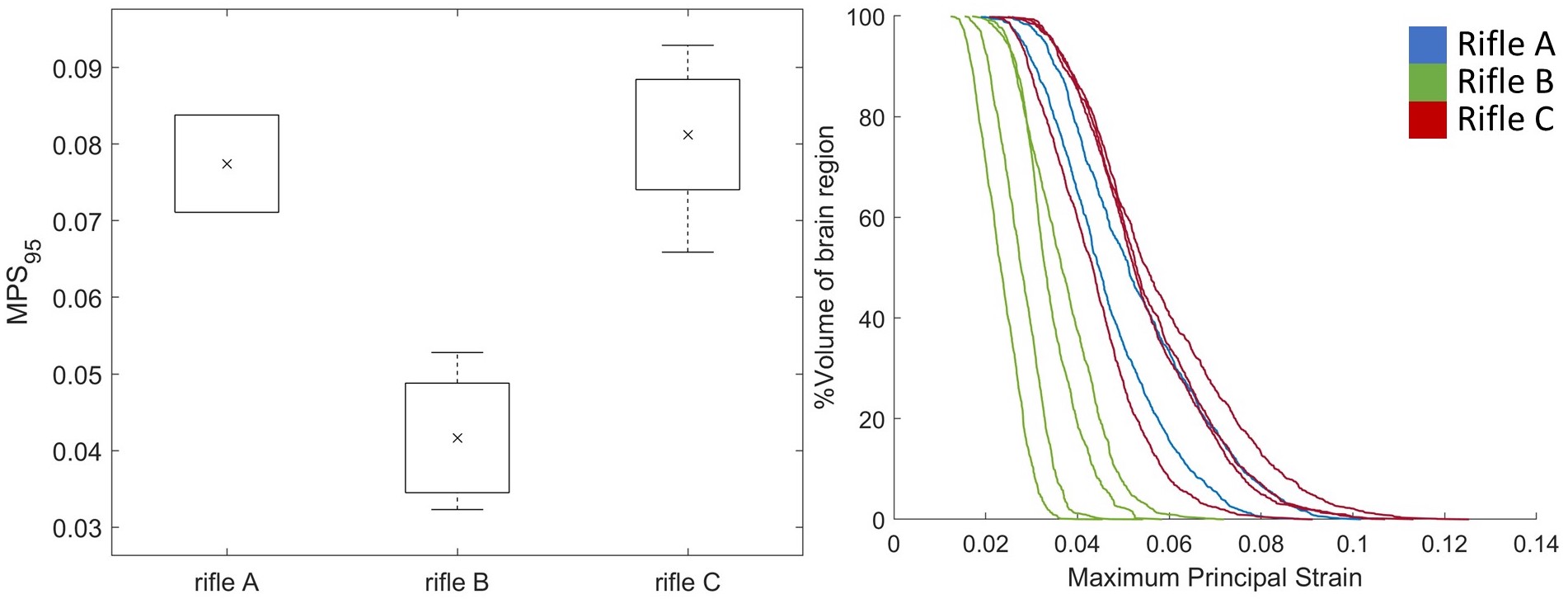


Supplementary Figure 19. Mean MPS_95_ and variation (A) and cumulative strain-volume curves (B) in the basal ganglia across all three rifles for volunteer 2.


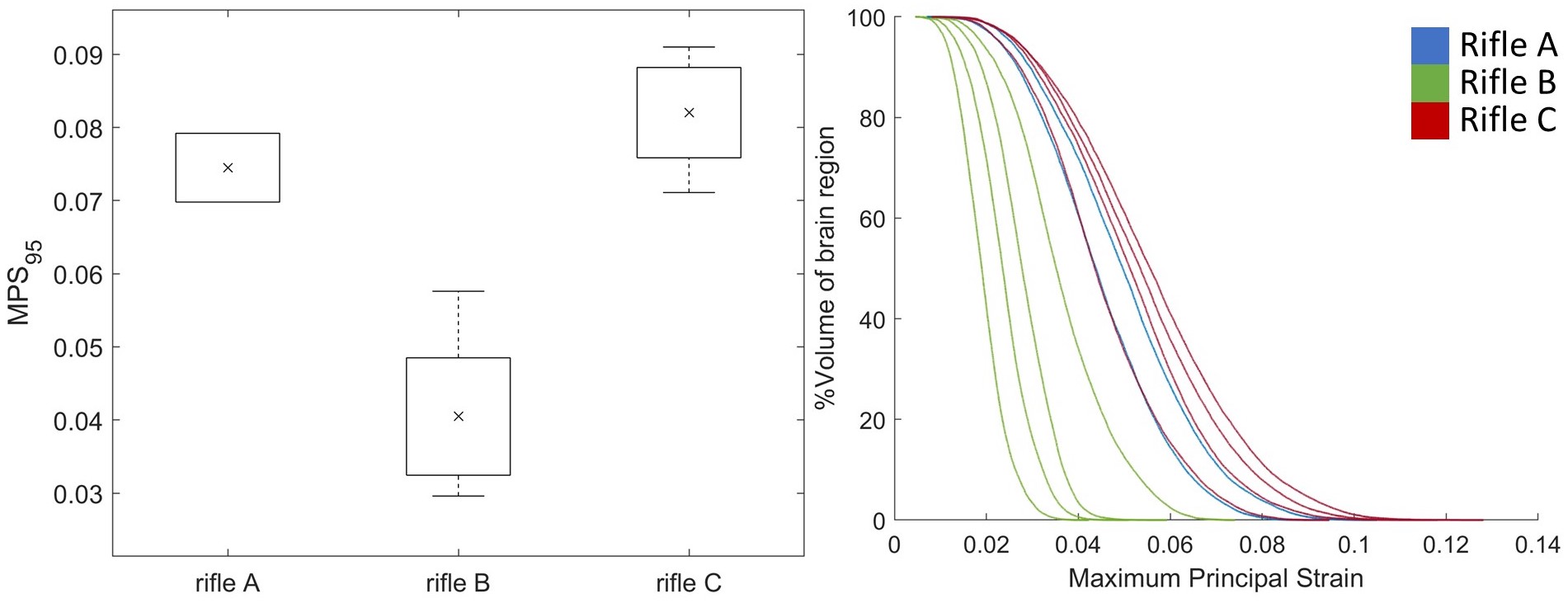


Supplementary Figure 20. Mean MPS_95_ and variation (A) and cumulative strain-volume curves (B) in the cerebrum white region across all three rifles for volunteer 2.

## Volunteer 3


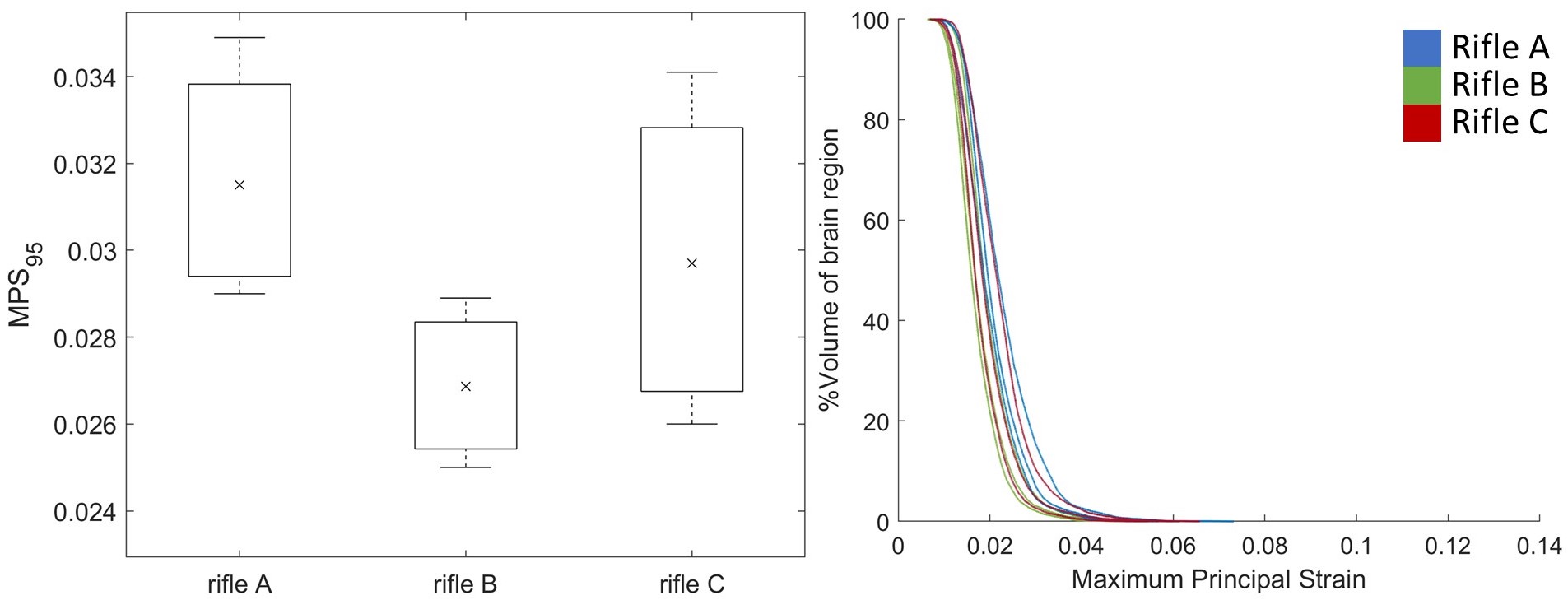


Supplementary Figure 21. Mean MPS_95_ and variation (A) and cumulative strain-volume curves (B) in the cerebellum across all three rifles for volunteer 3.


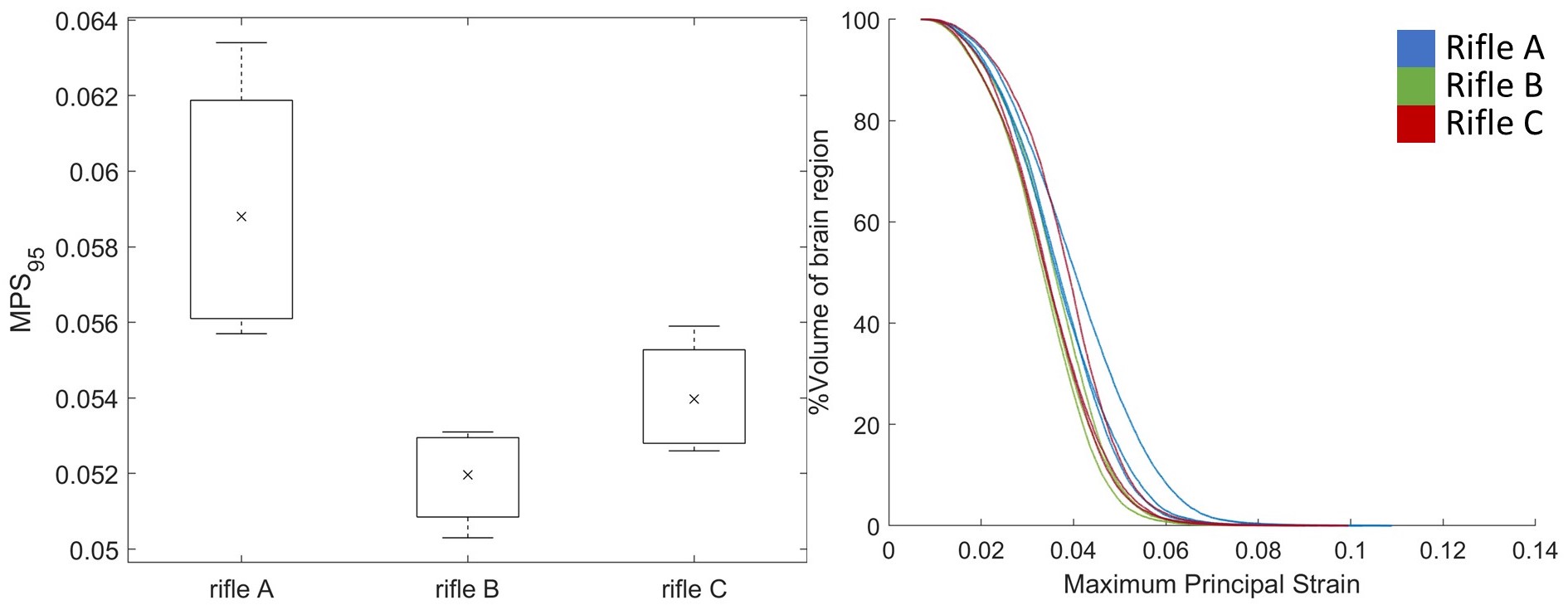


Supplementary Figure 22. Mean MPS_95_ and variation (A) and cumulative strain-volume curves (B) in the cerebrum gray region across all three rifles for volunteer 3.


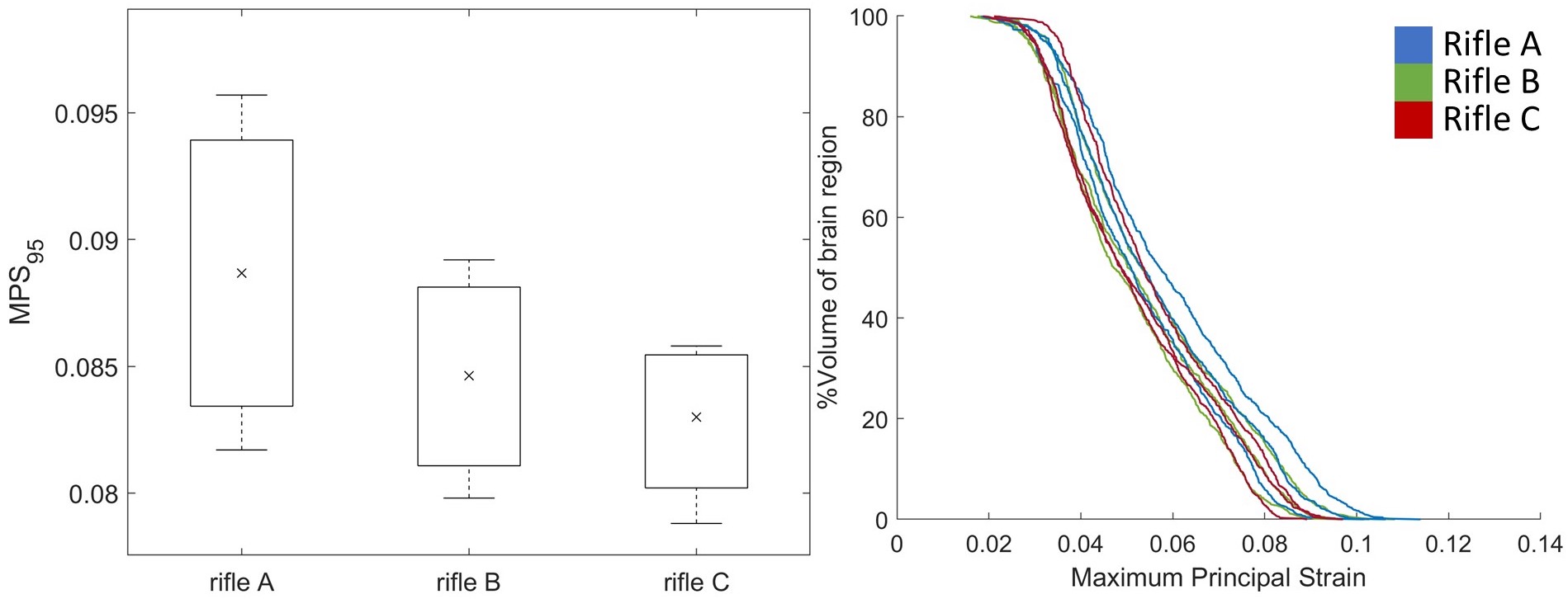


Supplementary Figure 23. Mean MPS_95_ and variation (A) and cumulative strain-volume curves (B) in the corpus callosum across all three rifles for volunteer 3.


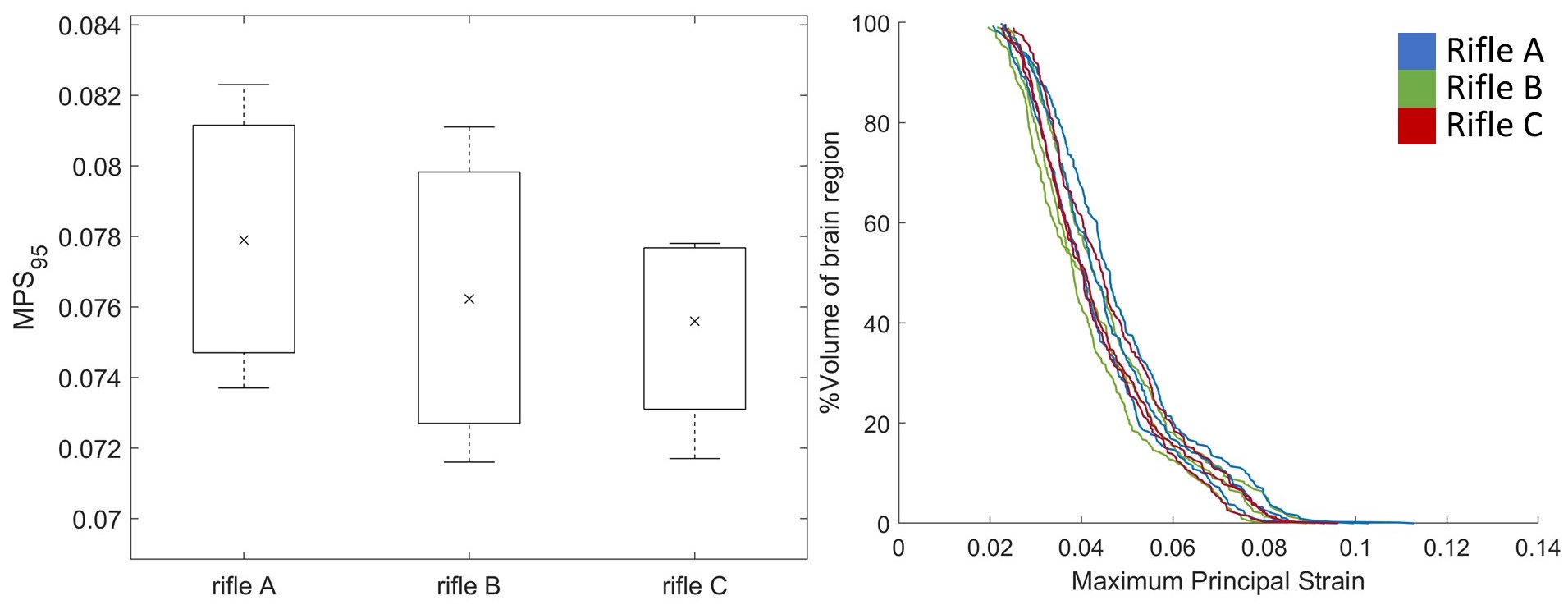


Supplementary Figure 24. Mean MPS_95_ and variation (A) and cumulative strain-volume curves (B) in the thalamus across all three rifles for volunteer 3.


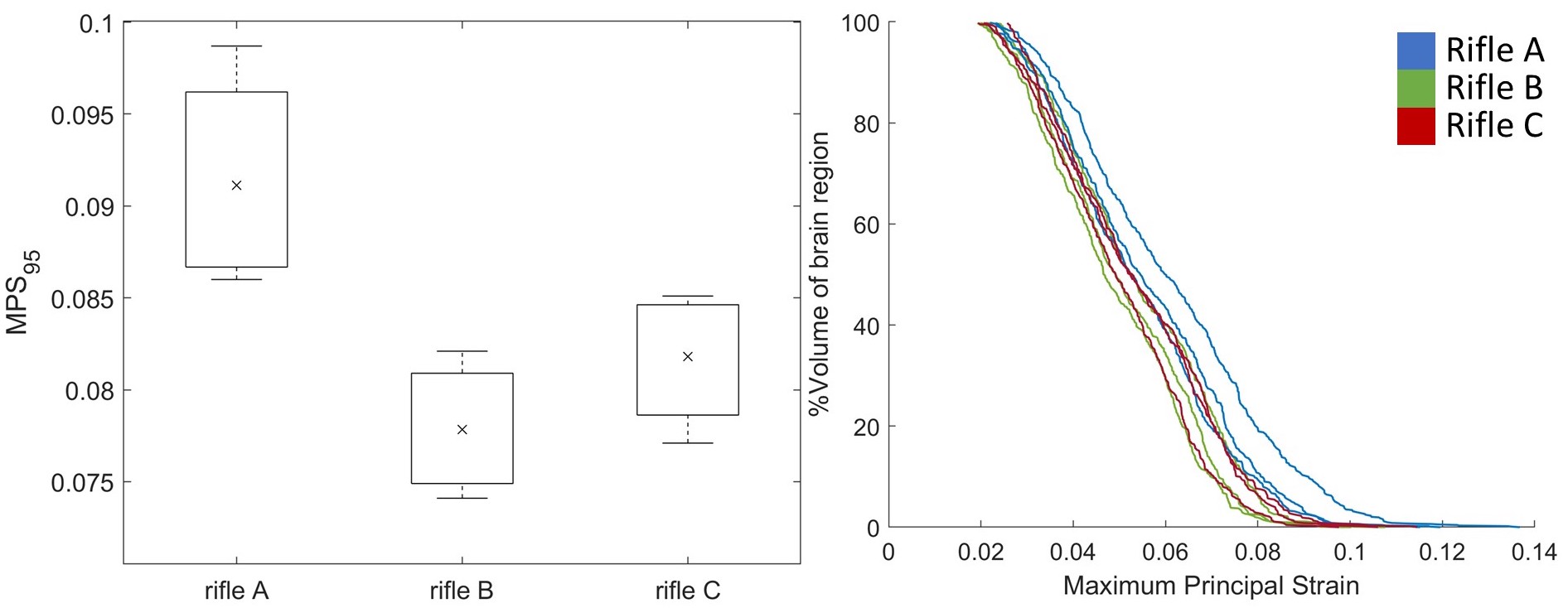


Supplementary Figure 25. Mean MPS_95_ and variation (A) and cumulative strain-volume curves (B) in the brainstem midbrain region across all three rifles for volunteer 3.


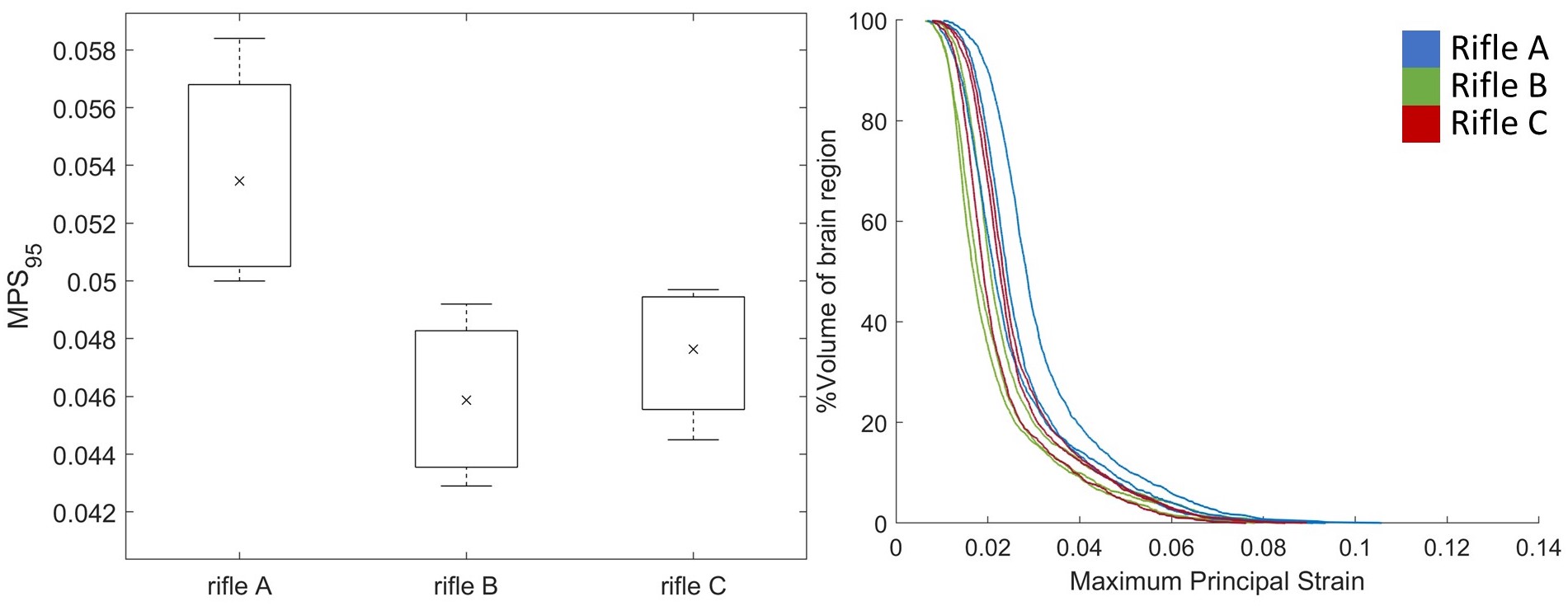


Supplementary Figure 26. Mean MPS_95_ and variation (A) and cumulative strain-volume curves (B) in the brainstem across all three rifles for volunteer 3.


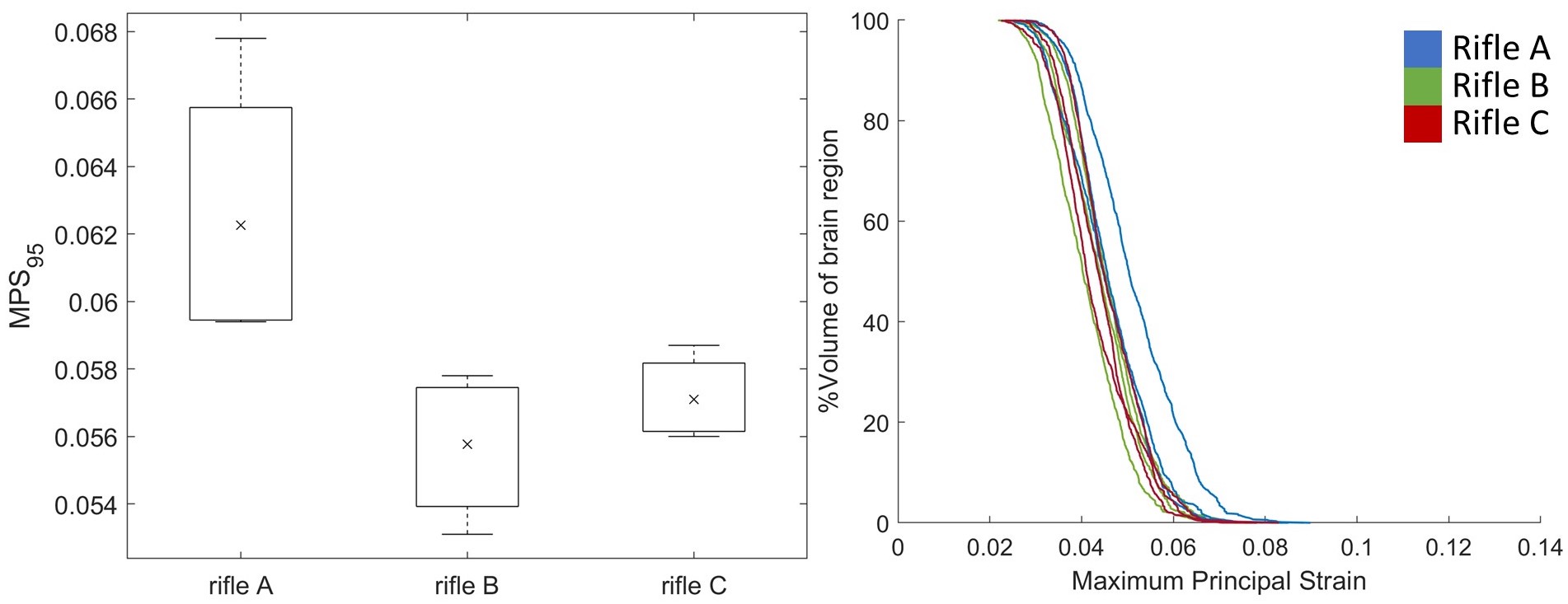


Supplementary Figure 27. Mean MPS_95_ and variation (A) and cumulative strain-volume curves (B) in the basal ganglia across all three rifles for volunteer 3.


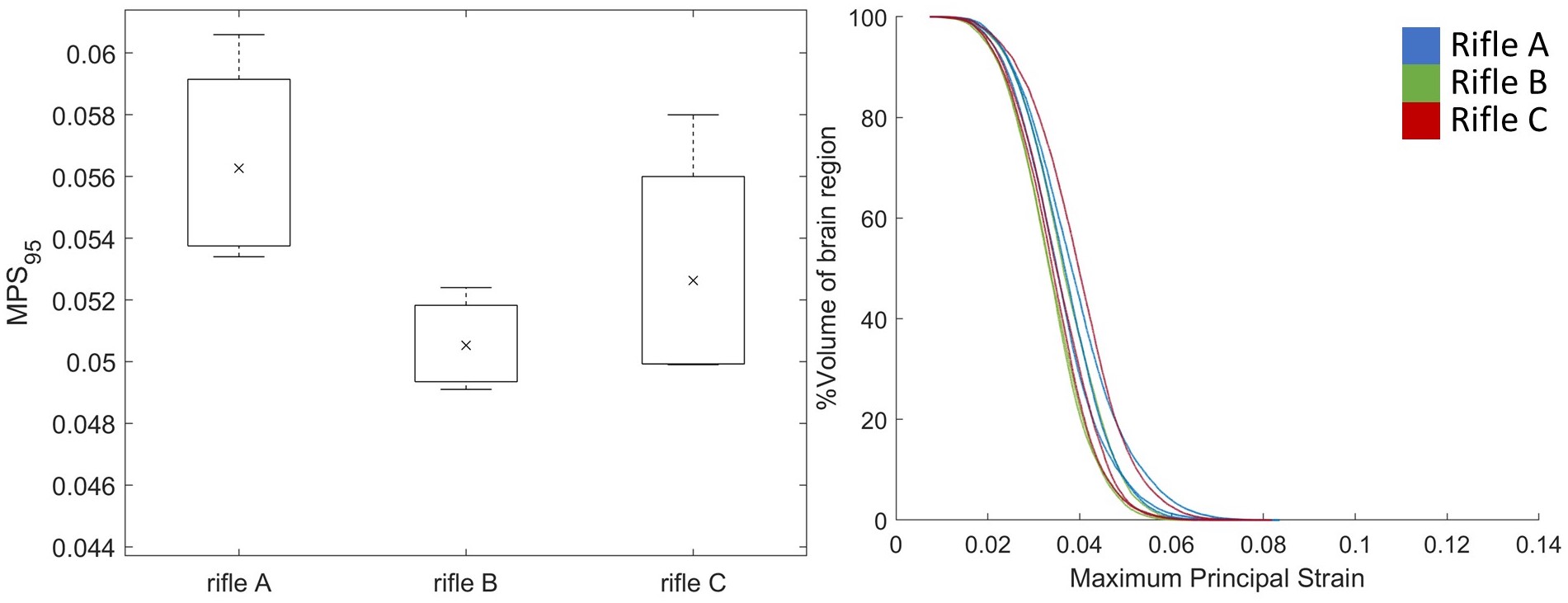


Supplementary Figure 28. Mean MPS_95_ and variation (A) and cumulative strain-volume curves (B) in the cerebrum white region across all three rifles for volunteer 3.

# ANOVA P-values for MPS_95_ and A_CSV_ of each of the eight individual brain regions for volunteers 1, 2 and 3

Supplementary Table 6: ANOVA P-values for MPS_95_ and A_CSV_ of individual brain regions for volunteer 1

|  | **Cerebellum** | | **Cerebrum Gray** | | **Corpus Callosum** | | **Thalamus** | |
| --- | --- | --- | --- | --- | --- | --- | --- | --- |
|  | **Mean MPS_95_** | **Mean A_CSV_** | **Mean MPS_95_** | **Mean A_CSV_** | **Mean MPS_95_** | **Mean A_CSV_** | **Mean MPS_95_** | **Mean A_CSV_** |
| **rifle A** | 0.0166 | 0.0112 | 0.0378 | 0.0256 | 0.0457 | 0.0313 | 0.0392 | 0.0250 |
| **rifle B** | 0.0150 | 0.0103 | 0.0331 | 0.0219 | 0.0420 | 0.0278 | 0.0371 | 0.0229 |
| **rifle C** | 0.0197 | 0.0133 | 0.0404 | 0.0264 | 0.0567 | 0.0360 | 0.0482 | 0.0297 |
| **P-value** | 0.0003 | 0.0002 | 0.0087 | 0.0090 | 0.0021 | 0.0033 | 0.0086 | 0.0023 |
| **significantly different?** | yes | yes | yes | yes | yes | yes | yes | yes |
|  | **Brainstem Midbrain** | | **Brainstem** | | **Basal Ganglia** | | **Cerebrum white** | |
|  | **Mean MPS_95_** | **Mean A_CSV_** | **Mean MPS_95_** | **Mean A_CSV_** | **Mean MPS_95_** | **Mean A_CSV_** | **Mean MPS_95_** | **Mean A_CSV_** |
| **rifle A** | 0.0537 | 0.0321 | 0.0264 | 0.0156 | 0.0389 | 0.0280 | 0.0365 | 0.0249 |
| **rifle B** | 0.0447 | 0.0269 | 0.0237 | 0.0131 | 0.0321 | 0.0231 | 0.0335 | 0.0223 |
| **rifle C** | 0.0563 | 0.0349 | 0.0313 | 0.0173 | 0.0406 | 0.0307 | 0.0403 | 0.0265 |
| **P-value** | 0.0202 | 0.0019 | 0.0008 | 0.0006 | 0.0097 | 0.0002 | 0.0140 | 0.0173 |
| **significantly different?** | yes | yes | yes | yes | yes | yes | yes | yes |

Supplementary Table 7: ANOVA P-values for MPS_95_ and A_CSV_ of individual brain regions for volunteer 2

|  | **Cerebellum** | | **Cerebrum Gray** | | **Corpus Callosum** | | **Thalamus** | |
| --- | --- | --- | --- | --- | --- | --- | --- | --- |
|  | **Mean MPS_95_** | **Mean A_CSV_** | **Mean MPS_95_** | **Mean A_CSV_** | **Mean MPS_95_** | **Mean A_CSV_** | **Mean MPS_95_** | **Mean A_CSV_** |
| **rifle A** | 0.0373 | 0.0243 | 0.0681 | 0.0432 | 0.0821 | 0.0573 | 0.0791 | 0.0503 |
| **rifle B** | 0.0206 | 0.0135 | 0.0411 | 0.0267 | 0.0559 | 0.0361 | 0.0503 | 0.0311 |
| **rifle C** | 0.0395 | 0.0252 | 0.0745 | 0.0469 | 0.0915 | 0.0625 | 0.0921 | 0.0569 |
| **P-value** | 0.0030 | 0.0052 | 0.0027 | 0.0023 | 0.0074 | 0.0029 | 0.0044 | 0.0042 |
| **significantly different?** | yes | yes | yes | yes | yes | yes | yes | yes |
|  | **Brainstem Midbrain** | | **Brainstem** | | **Basal Ganglia** | | **Cerebrum white** | |
|  | **Mean MPS_95_** | **Mean A_CSV_** | **Mean MPS_95_** | **Mean A_CSV_** | **Mean MPS_95_** | **Mean A_CSV_** | **Mean MPS_95_** | **Mean A_CSV_** |
| **rifle A** | 0.0683 | 0.0450 | 0.0410 | 0.0261 | 0.0775 | 0.0499 | 0.0745 | 0.0473 |
| **rifle B** | 0.0586 | 0.0353 | 0.0310 | 0.0163 | 0.0416 | 0.0307 | 0.0405 | 0.0268 |
| **rifle C** | 0.0733 | 0.0486 | 0.0402 | 0.0248 | 0.0813 | 0.0531 | 0.0820 | 0.0517 |
| **P-value** | 0.0964 | 0.0274 | 0.0859 | 0.0337 | 0.0019 | 0.0032 | 0.0015 | 0.0015 |
| **significantly different?** | no | yes | no | yes | yes | yes | yes | yes |

Supplementary Table 8: ANOVA P-values for MPS_95_ and A_CSV_ of individual brain regions for volunteer 3

|  | **Cerebellum** | | **Cerebrum Gray** | | **Corpus Callosum** | | **Thalamus** | |
| --- | --- | --- | --- | --- | --- | --- | --- | --- |
|  | **Mean MPS_95_** | **Mean A_CSV_** | **Mean MPS_95_** | **Mean A_CSV_** | **Mean MPS_95_** | **Mean A_CSV_** | **Mean MPS_95_** | **Mean A_CSV_** |
| **rifle A** | 0.0315 | 0.0197 | 0.0588 | 0.0358 | 0.0887 | 0.0534 | 0.0779 | 0.0445 |
| **rifle B** | 0.0269 | 0.0211 | 0.0520 | 0.0382 | 0.0846 | 0.0566 | 0.0762 | 0.0458 |
| **rifle C** | 0.0297 | 0.0181 | 0.0540 | 0.0344 | 0.0830 | 0.0534 | 0.0756 | 0.0433 |
| **P-value** | 0.2681 | 0.2224 | 0.0526 | 0.1355 | 0.4556 | 0.4094 | 0.7908 | 0.5417 |
| **significantly different?** | no | no | no | no | no | no | no | no |
|  | **Brainstem Midbrain** | | **Brainstem** | | **Basal Ganglia** | | **Cerebrum white** | |
|  | **Mean MPS_95_** | **Mean A_CSV_** | **Mean MPS_95_** | **Mean A_CSV_** | **Mean MPS_95_** | **Mean A_CSV_** | **Mean MPS_95_** | **Mean A_CSV_** |
| **rifle A** | 0.0911 | 0.0524 | 0.0534 | 0.0252 | 0.0623 | 0.0442 | 0.0563 | 0.0363 |
| **rifle B** | 0.0778 | 0.0571 | 0.0459 | 0.0285 | 0.0558 | 0.0476 | 0.0505 | 0.0371 |
| **rifle C** | 0.0818 | 0.0511 | 0.0477 | 0.0227 | 0.0571 | 0.0436 | 0.0526 | 0.0346 |
| **P-value** | 0.0462 | 0.1162 | 0.0852 | 0.0871 | 0.0983 | 0.2023 | 0.2210 | 0.4409 |
| **significantly different?** | yes | no | no | no | no | no | no | no |

# Area under the cumulative strain-volume curves (A_CSV_) measured and plotted in a bar chart format for each brain region while volunteers fired rifles A, B and C.


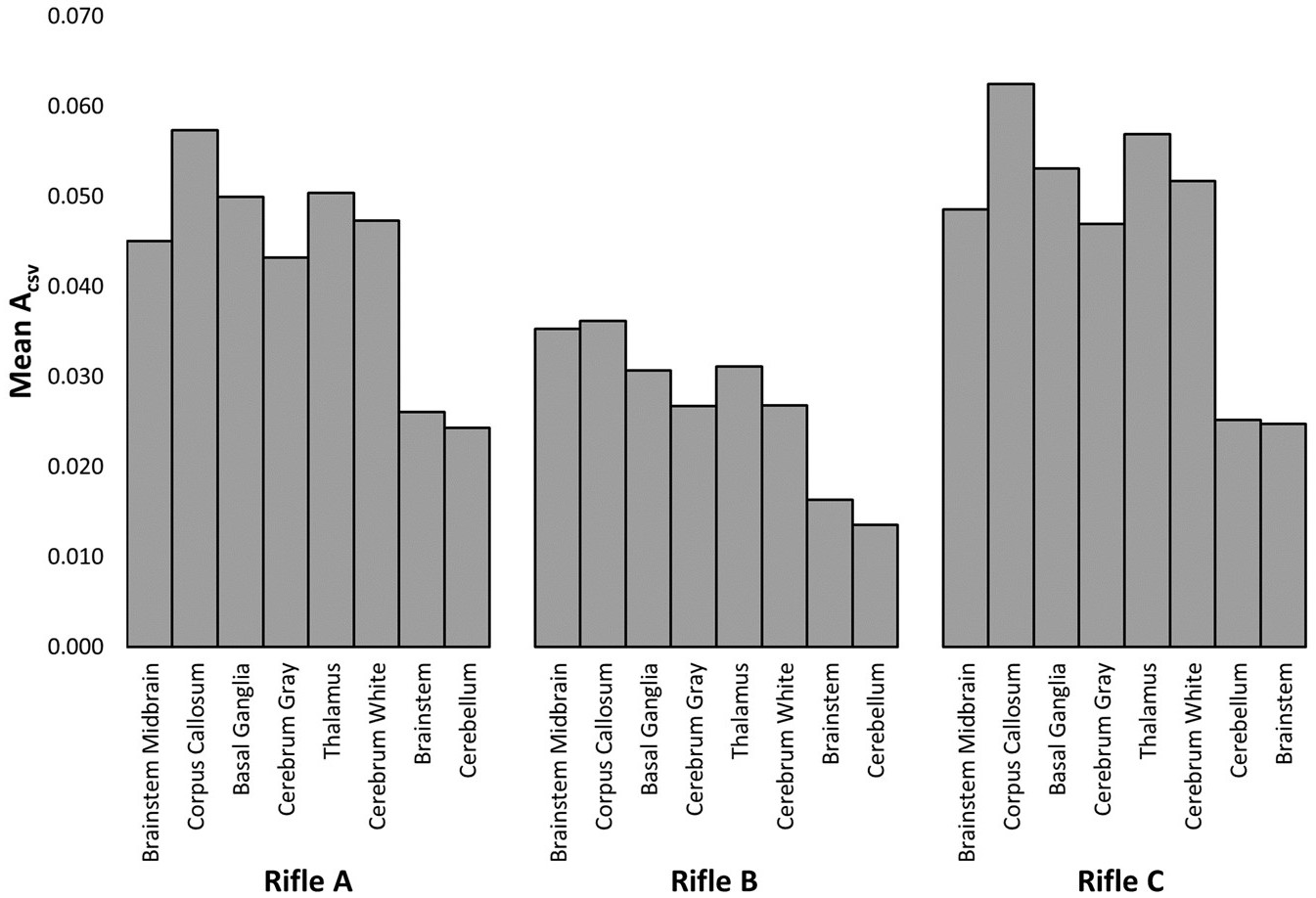


Supplementary Figure 29. The area under the cumulative strain-volume curves (A_CSV_) measured for each brain region while volunteer 2 fired rifles A, B and C.


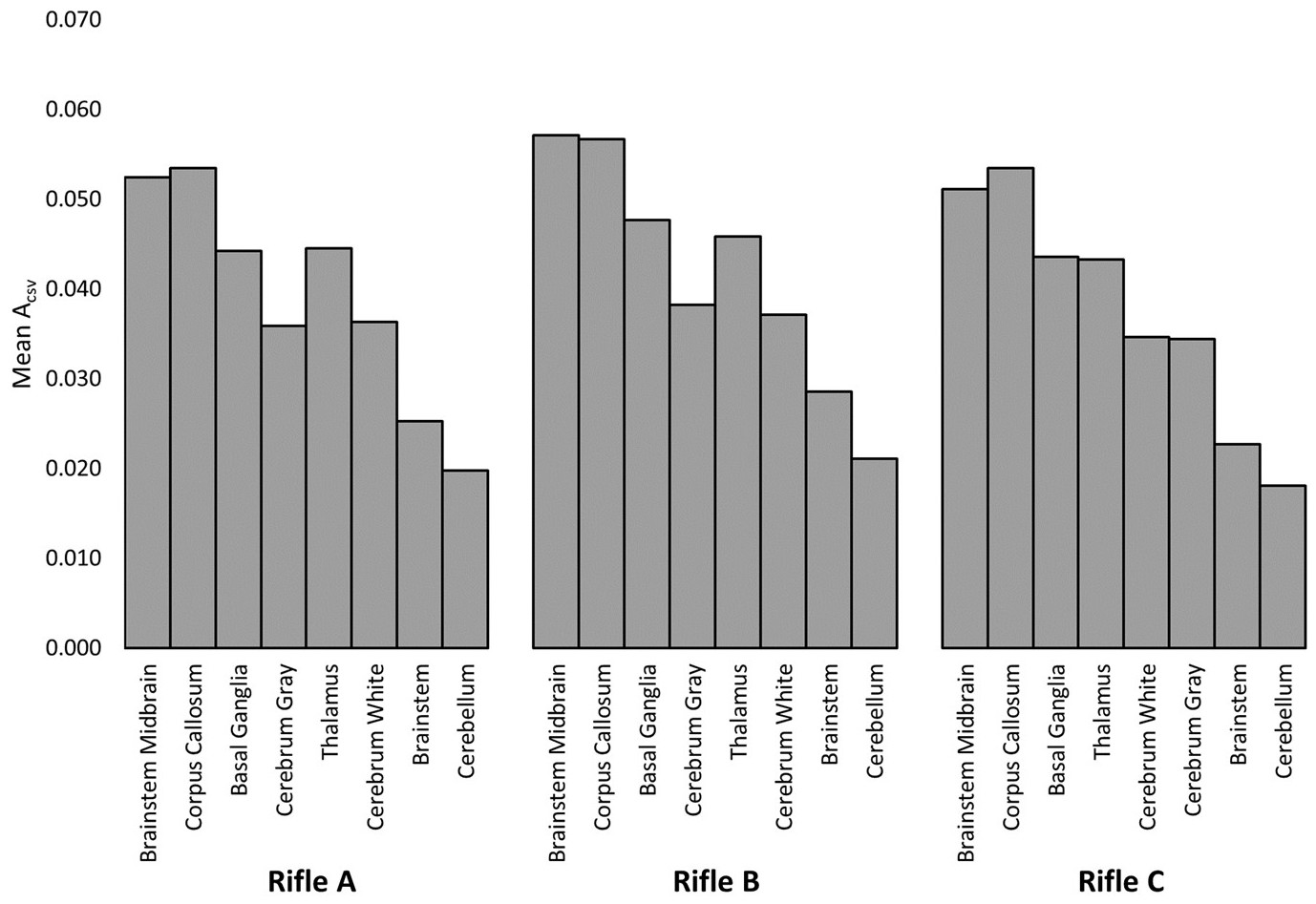


Supplementary Figure 30. The area under the cumulative strain-volume curves (A_CSV_) measured for each brain region while volunteer 3 fired rifles A, B and C.
